# Co‐occurrence of *BAP1* and *SF3B1* mutations in uveal melanoma induces cellular senescence

**DOI:** 10.1002/1878-0261.13128

**Published:** 2021-11-12

**Authors:** Le Yu, Dan Zhou, Guiming Zhang, Zhonglu Ren, Xin Luo, Peng Liu, Steven W. Plouffe, Zhipeng Meng, Toshiro Moroishi, Yilei Li, Yiyue Zhang, Joan Heller Brown, Shuwen Liu, Kun‐Liang Guan

**Affiliations:** ^1^ School of Pharmaceutical Sciences Southern Medical University Guangzhou China; ^2^ College of Medical Information Engineering Guangdong Pharmaceutical University Guangzhou China; ^3^ Medicinal Information and Real World Engineering Technology Center of Universities Guangzhou China; ^4^ Institutes of Biomedical Sciences Fudan University Shanghai China; ^5^ Department of Pharmacology and Moores Cancer Center University of California San Diego CA USA; ^6^ Center for Metabolic Regulation of Healthy Aging Faculty of Life Sciences Kumamoto University Japan; ^7^ Department of Cell Signaling and Metabolic Medicine Faculty of Life Sciences Kumamoto University Japan; ^8^ Precursory Research for Embryonic Science and Technology Japan Science and Technology Agency Saitama Japan; ^9^ Department of Pharmacy Nanfang Hospital Southern Medical University Guangzhou China; ^10^ Division of Cell Development and Integrative Biology School of Medicine South China University of Technology Guangzhou China

**Keywords:** BAP1, mutually exclusive pattern, recurrent mutations, senescence, SF3B1, uveal melanoma

## Abstract

Uveal melanoma (UM) is the most common intraocular tumor in adults. Recurrent mutations in BRCA1‐associated protein 1 (*BAP1*) and splicing factor 3B subunit 1 (*SF3B1*) display a mutually exclusive pattern in UM, but the underlying mechanism is unknown. We show that combined BAP1 deficiency and *SF3B1* hotspot mutation lead to senescence and growth arrest in human UM cells. Although p53 protein expression is induced, deletion of *TP53* (encoding p53) only modestly rescues the observed senescent phenotype. UM cells with BAP1 loss or SF3B1 mutation are more sensitive to chemotherapeutic drugs compared with their isogenic parental cells. Transcriptome analysis shows that DNA‐repair genes are downregulated upon co‐occurrence of BAP1 deletion and SF3B1 mutation, thus leading to impaired DNA damage response and the induction of senescence. The co‐occurrence of these two mutations reduces invasion of UM cells in zebrafish xenograft models and suppresses growth of melanoma xenografts in nude mice. Our findings provide a mechanistic explanation for the mutual exclusivity of *BAP1* and *SF3B1* mutations in human UM.

## Graphical abstract

The transcriptional suppression of DNA‐repair genes derived from co‐occurrence of BRCA‐1‐associated protein 1 (BAP1) deficiency and splicing factor 3B subunit 1 (SF3B1) mutation (R625H) impairs cells’ capacity to buffer endogenous DNA damage and consequently leads to DNA damage and senescence. Our findings provide a functional explanation for the observed mutual exclusivity of *BAP1* and *SF3B1* mutations in uveal melanoma.

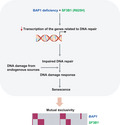

**Abbreviations**
53BP1: p53‐binding protein
BAP1: BRCA1‐associated protein 1
BNLM: blue nevus‐like melanoma
CM: cutaneous melanoma
CRISPR: clustered regularly interspaced short palindromic repeats
DEGs: differentially expressed genes
EIF1AX: eukaryotic translation initiation factor 1A, X chromosomal
H2AK119ub1: ubiquitinated histone 2A at lysine 119
KEGG: Kyoto Encyclopedia of Genes and Genomes
KO: knockout
PARP: poly (ADP‐ribose) polymerase
PLMT: primary leptomeningeal melanocytic tumor
PR‐DUB: polycomb repressive deubiquitinase
SA‐β‐gal: senescence‐associated acidic β‐galactosidase
SF3B1: splicing factor 3B subunit 1
UM: uveal melanoma

## 1 Introduction

Uveal melanoma (UM), the most common primary intraocular tumor in adults, originates from melanocytes in the anterior (iris) or posterior uveal tract (choroid and ciliary body) [1, 2]. Approximately half of UM patients develop metastasis, with about 90% metastasis in the liver. Once metastasized, there are no effective therapies at present, with an overall survival of only 6 months [3].

UM is molecularly distinct from cutaneous melanoma (CM) and shows a different pattern of driver mutations. Instead of the *BRAF* or *NRAS* mutations common in CM, UM harbors mutually exclusive somatic mutations in the G protein‐coupled receptor (GPCR) signal transducer *GNAQ* and *GNA11* (encoding Gq and G11, respectively) (92.5%), the GPCR *CYSLTR2* (4%), and downstream effector *PLCB4* (2.5%) genes. CYSLTR2 is known to couple to Gq/11 to activate PLCβ4; thus, these genes act in the same signaling pathway. These observations suggest that constitutively active Gq signaling, particularly the Gq/11 coupled, plays a central role in driving tumorigenesis in virtually all UM [4]. We have previously shown that Gq/11 mutations act through the Hippo pathway to induce tumorigenesis [5]. In contrast, only a minor fraction (5%) of UM cases carries *BRAF* mutations [6]. Other common UM mutations include BRCA1‐associated protein 1 (*BAP1*), splicing factor 3B subunit 1 (*SF3B1*), and eukaryotic translation initiation factor 1A, X chromosomal (*EIF1AX*) [7, 8]. Loss‐of‐function mutations in the *BAP1* tumor suppressor gene are strongly correlated with metastasis [9, 10], while mutations in *SF3B1* and *EIF1AX* are associated with intermediate and low risk, respectively [4, 11]. Interestingly, *BAP1* and *SF3B1* mutations are also mutually exclusive in UM [4]. However, the molecular insights underlying this mutually exclusive mutation pattern are unknown.

Notably, blue nevus‐like melanoma (BNLM) and primary leptomeningeal melanocytic tumor (PLMT) have genetic mutations similar to UM, but distinct from CM. Blue nevi are common dermal melanocyte proliferations and largely benign; however, low‐frequency malignant transformation gives rise to BNLM. Mutually exclusive mutations in *GNAQ* or *GNA11* are most common in blue nevi while low‐frequency mutations in either *CYSLTR2* or *PLCB4* also exist [12, 13]. Interestingly, *BAP1* deletions and *SF3B1* hotspot mutations are associated with BNLM, indicating their positive role in malignancy. PLMT occurs in the central nervous system and displays genetic mutations similar to UM, including high‐frequency mutations in *GNAQ/11* and low‐frequency mutations in *CYSLTR2* and *PLCB4* in mutually exclusive manner. Furthermore, mutations in *BAP1*, *SF3B1*, and *EIF1AX* are also commonly observed in PLMT [14].

The *BAP1* gene encodes a ubiquitin carboxyl terminal hydrolase with deubiquitinase activity [15]. BAP1 is predominantly in the nucleus where it interacts with transcription‐related proteins such as FOXK1/2, YY1, HCF1, and OGT1 to regulate the expression of target genes [16, 17, 18]. BAP1 and ASXL1/2/3 form the polycomb repressive deubiquitinase (PR‐DUB) complex, which mainly functions to remove monoubiquitin from ubiquitinated histone 2A at lysine 119 (H2AK119ub1) [19]. It has been shown that the delicate balance between H2A ubiquitination and deubiquitination helps to fine‐tune gene expression [20]. Accordingly, BAP1 influences a wide array of cellular functions, such as transcriptional regulation and the DNA damage response [21]. Previous work has shown that knockdown of BAP1 in UM cell lines by siRNA/shRNA leads to the loss of melanocyte identity and acquisition of a de‐differentiated, stem‐like phenotype which conceivably contributes to their pro‐metastatic behavior [9, 22].

The *SF3B1* gene encodes subunit 1 of the splicing factor 3b protein complex, which is essential in pre‐mRNA splicing [23]. *SF3B1* mutations are typically heterozygous and cluster at hotspots, a mutation pattern that is characteristic for oncogenes. SF3B1 K700 mutation is common in hematological malignancies, while mutations in R625, and less frequently in K666, are found in UM [7, 8, 24, 25]. SF3B1 mutations have been reported to induce cryptic 3’ splice site selection through alternative branchpoint usage thus affecting a wide range of primary transcripts [26].

Integrative analysis showed that *BAP1*‐ and *SF3B1*‐mutant UM have distinct genomic aberrations, transcriptional features, and clinical outcomes, whereas *GNAQ*‐ and *GNA11*‐mutant UM do not show such difference [4]. The mutual exclusivity for *GNAQ* and *GNA11* mutations in UM can be explained by their similar functions in cellular signaling with mutation of one gene relieving selective pressure of mutating the other. Unlike *GNAQ* and *GNA11*, we hypothesize that *BAP1* and *SF3B1* mutations affect different cellular processes and their concurrent mutations may induce synthetic unfitness to cells, thus displaying mutual exclusivity in their mutation spectrum. Here, we determined the effect of co‐occurrence of BAP1 and SF3B1 mutations using cell models expressing single or double mutations simultaneously. This report reveals a molecular mechanism accounting for the observed mutual exclusivity of *BAP1* and *SF3B1* mutations in UM patients.

## 2 Materials and methods

### 2.1 Cell Lines, culture procedures, and chemicals

UM cell lines (92.1, Mel202, Mel270, OMM2.2, OMM2.3, OMM2.5, OMM1, OCM1, Mel285, and Mel290), provided by Dr. Martine Jager (Leiden University), were maintained as previously described [5]. Y‐MESO‐14 was a gift from Dr. Yoshitaka Sekido (Aichi Medical University), and NCI‐H2373 was purchased from ATCC. These two mesothelioma cell lines were maintained as previously described [27]. Human embryonic kidney 293T cells were from ATCC and cultured in Dulbecco’s modified Eagle’s medium with 10% fetal bovine serum. All media supplemented with 50 μg·mL^−1^ penicillin/streptomycin. Cells were maintained at 37 °C with 5% CO2 and confirmed to be free of mycoplasma. Olaparib (PARP inhibitor, TargetMol) and temozolomide (alkylating agent, Sigma‐Aldrich, Shanghai, China) were pre‐prepared as stock solution in DMSO and used at concentrations indicated in figures.

### 2.2 SA‐β‐Galactosidase staining

SA‐β‐gal (senescence‐associated β‐galactosidase) assay was performed in a 6‐well plate using a staining kit (Sigma‐Aldrich). Cells were washed with PBS, then fixed, and stained following the manufacturer’s instructions.

### 2.3 EdU staining

5‐Ethynyl‐2’‐deoxyuridine (EdU) staining was performed using Click‐iT^TM^ Plus EdU kit (Invitrogen, Carlsbad, CA, USA) per manufacturer protocol. The proliferation capacity was analyzed by measuring the percentage of EdU‐positive cells in DAPI‐stained cells.

### 2.4 Cell cycle analysis

Cells were fixed with ice‐cold 75% ethanol in PBS and stained using propidium iodide. The distribution of cells in different phases of the cell cycle was analyzed by flow cytometry (BD FACSCanto II).

### 2.5 Elisa

IL‐6 and TNF‐α in cell culture supernatant were measured by Elisa, using assay kits (Sino Biological, Beijing, China) in accordance with the manufacturer’s instructions. The data were normalized to cell numbers.

### 2.6 Colony formation assay

Cells (2000 cells per well) were seeded in six‐well plate. Colonies were stained using 0.005% crystal violet in 5% methanol and counted 10–14 days after plating.

### 2.7 PD calculation

Population doublings (PD) were calculated as described previously [28]. Cells infected with *BAP1* sgRNA g2 or control viruses were selected with puromycin for 3 days and recovered in fresh medium. The day when cells were plated in 12‐well plates at 4 × 10^4^ per well in triplicates (7 days postinfection) was defined as day 0. Every 4 days, cells were harvested and counted. At each split, 4 × 10^4^ cells were replated to fresh plates and allowed to grow until next split. PD were calculated with the formula PD = log(N2/N1)/log2, where N1 is the number of cells plated and N2 is that of recovered.

### 2.8 Cell viability assay

Cells were seeded in 96‐well plates. Seeded cell numbers: Mel202 (4000 cells), OMM2.3 (1500 cells), OCM1 (900 cells). After attachment, cells were treated with olaparib or temozolomide at the indicated concentrations for 6 days. MTT was then added, and the cells were further incubated in the presence of 0.5 mg·mL^−1^ MTT for 3 h. The optical density was measured spectrophotometrically at 570 nm in a multiwall plate reader (Bio‐Rad Laboratories, Hercules, CA, USA). Each condition consisted of, at least, three replicate wells, and data were expressed as the percentage of survival of vehicle control (DMSO) cells.

### 2.9 Anchorage‐independent growth assay

Each six‐well plate was coated with 1.5 mL of bottom agar (0.5% Difco agar noble in normal culture medium). Mel202 (10 000) or OMM2.3 (6000) was suspended in 1.5 mL of top agar (0.3% Difco agar noble) into each well. Cells were treated with 200 ng·mL^−1^ doxycycline for 6 days to trigger CRISPR‐mediated gene editing and then incubated in normal culture medium for approximately 2 weeks. Colonies were stained using 0.005% crystal violet.

### 2.10 Transwell cell migration assay

Cell migration assays were performed using transwell plates (8 μm pore size polycarbonate membrane) (Corning). The bottom membrane was pre‐coated with fibronectin (20 μg·mL^−1^). Cells were seeded (4 × 10^4^ cells) into the upper chamber of the insert in serum‐free media. Lower chamber was filled with complete medium. After 24 h, cells were fixed and stained with 0.1% crystal violet. Cells in the upper chamber were carefully removed, and cells that migrated through the filter were assessed by photography.

### 2.11 Generation of knockout cells

CRISPR‐Cas9 system was used to delete genes in Mel270, OMM2.3, Mel202, and OCM1 cells. The plasmid px459 v2 and lentiCRISPR v2 were provided by Dr. Feng Zhang (Addgene plasmids #62988 and 52961) [29]. Gene‐specific sgRNAs were designed using the CRISPR design tool at http://crispr.mit.edu. Cells were transfected, selected with puromycin for 3 days, and single‐cell sorted by FACs (UCSD; Human Embryonic Stem Cell Core, BDInflux) into 96‐well plate format. Single clones were expanded and screened by protein immunoblotting and confirmed by genomic sequencing. For inducible BAP1 knockout experiment, a two vector (lentiGuide‐Hygro backbone) system was used. Cas9‐Mel202, Cas9‐OMM2.3, and Cas9‐OCM1 cells were generated by lentiviral transduction of the doxycycline‐inducible FLAG‐Cas9 vector pCW‐Cas9 (Addgene plasmid #50661). Following a 3‐day selection with 1 μg·mL^−1^ puromycin, the cells were sorted onto 96‐well plates with only one cell in each well. The level of FLAG‐Cas9 expression in the presence and absence of 200 ng·mL^−1^ doxycycline was analyzed for many clones by western blotting of FLAG‐tag (Sigma‐Aldrich). Subsequently, a single colony with strong induction and minimal background level of Cas9 expression was selected from each cell line. To generate cell lines stably expressing sgRNAs targeting *BAP1* gene, the above clones with inducible FLAG‐Cas9 were transduced with a lentiviral pGuide‐Puro‐BAP1 vector (Addgene plasmid #52963), which was modified to confer resistance to hygromycin, and selected with hygromycin (500 μg·mL^−1^) for 5 days. To induce gene knockout, Cas9 expression was induced by the addition of doxycycline (200 ng·mL^−1^). Guide sequences used in the study are listed in Table S1.

### 2.12 Retroviral infection and mutagenesis

pQCXIH‐HA‐RNaseH1 and pQCXIH‐BAP1 were purchased from Youbio Technology Co., Ltd. pQCXIH‐Flag‐SF3B1 and pRetroX‐Tight‐HA‐SF3B1‐GFP were obtained by subcloning the human *SF3B1* cDNA of pENTR4‐SF3B1 vector (provided by Dr. Manoj Pillai in Yale University) in the pQCXIH (Clontech plasmid #631516) and pRetroX‐Tight‐GFP expression vectors, respectively. pRetroX‐Tight‐GFP was derived from pRetroX‐Tight‐Pur (Clontech plasmid #632104) by replacing the puromycin resistance gene with coding sequence for GFP gene. Retroviral packaging and infection were performed as described previously [5]. SF3B1 site‐directed mutagenesis was carried out using Q5 Hot Start High Fidelity DNA polymerase from New England Biolabs per manufacturer protocol. The mutagenic oligonucleotide primer pairs for substitution were as follows: SF3B1(R625H)‐forward: CGAGTATGTGCATAATACCACAGCTAG; SF3B1(R625H)‐reverse: TCCATGTTGTCAATATCTGG. The resulting construct was verified by Sanger sequencing. Gene overexpression was confirmed by immunoblot analysis.

### 2.13 Immunoblotting

Immunoblotting was performed using a standard protocol. The detailed information of the antibodies is provided in Table S2.

### 2.14 Immunofluorescence

Cells were fixed in 4% paraformaldehyde/PBS for 15 min followed by permeabilization with 0.1% Triton X for 5 min. Cells were then blocked in 3% BSA for 1 hr and incubated overnight at 4 °C in primary antibodies diluted in 3% BSA. Secondary antibodies and phalloidin were diluted in 3% BSA and incubated for 90 min. Cells were covered with a drop of prolong gold antifade reagent with DAPI (Thermo Fisher Scientific, Waltham, MA, USA) for observation. Images were captured with a Nikon Eclipse Ti confocal microscope. Images depicted in figures were exported from NIS elements imaging software. Primary and secondary antibodies used for immunofluorescence are listed in Table S2.

### 2.15 RNA Interference

For inducible knockdown of BAP1, cells were infected with doxycycline‐inducible shRNAs for BAP1 (Sigma‐Aldrich, Cat#TRCN0000007374) using a lentiviral vector Tet‐pLKO‐puro (Addgene plasmid #21915). Duplex siRNAs targeting BAP1 (Sigma‐Aldrich, Cat#SASI_Hs01_00105395 and SASI_Hs01_00105396) were transfected into cells with HiPerFection Transfection Reagent (Qiagen, Hilden, Germany). Non‐targeting siRNAs (Sigma‐Aldrich, Cat#SIC001) were used as control.

### 2.16 Quantitative Real‐time PCR

Total RNA was extracted using RNeasy Plus Mini Kit from Qiagen according to the manufacturer’s instructions. RNA samples were reverse‐transcribed to cDNA using iScript cDNA synthetic Kit (BIO‐RAD) according to manufacturer’s protocol. Primer sequences were obtained from PrimerBank (http://pga.mgh.harvard.edu/primerbank/). Real‐time PCR was performed using KAPA SYBR FAST qPCR kit (Kapa Biosystems) and an Applied 7300 real‐time PCR system. The average cycle threshold (CT) values were determined. Target gene expression was normalized to housekeeping gene *GAPDH*.

### 2.17 Genomic DNA and cDNA sequencing

Genomic DNA was extracted from cells using PureLink Genomic DNA Mini Kit (Thermo Fisher Scientific) following manufacturer’s instructions. For analyzing *SF3B1* and *EIF1AX* mutations of UM cell lines, PCR was used to amplify exon 14 of *SF3B1* and exon 1 and exon 2 of *EIF1AX*. For analyzing genome modification by CRISPR‐Cas9, PCR primers were designed to span the Cas9 cleavage site. Indel mutations were analyzed through Sanger sequencing. For analyzing allele‐specific *SF3B1* gene editing by CRISPR‐Cas9, reverse transcription PCR was used to amplify exon 1‐16 of *SF3B1* cDNA, as previously described [30]. For analyzing allele‐specific *GNAQ* gene editing, reverse transcription PCR products were sequenced directly or after TA cloning.

### 2.18 RNA sequencing and bioinformatics analysis

Total RNAs were extracted by TRIzol (Thermo Fisher Scientific) from OMM2.3 and Mel202 cells transfected with lentiCRISPR‐Cas9‐expressing empty vector or *BAP1* sgRNA (g2). Three replicates for each sample were generated and analyzed. A total amount of 1 μg RNA per sample was used for the RNA sample preparations. RNA was then sent to a sequencing company (Novogene) for sequencing. Read quality was evaluated using FastQC. Read pseudo‐alignment to the reference transcriptome (hg38) and their counting were performed using Kallisto [31]. The abundance values were then imported into R platform using tximport package [32]. DEGs (differentially expressed genes) were identified by DESeq2 package [33]. Genes with adjusted *P* < 0.05 were considered as significant DEGs unless otherwise stated. KEGG enrichment analysis of DEGs was performed using the online database KOBAS3.0.

### 2.19 Animal work

All experiments involving zebrafish and nude mice were performed in accordance with the guidelines by the Institutional Animal Care and Use Committee of Southern Medical University (2015‐0056).

Wild‐type AB zebrafish were obtained from China Zebrafish Resource Center (Wuhan, China). Zebrafish embryos were raised at 28 °C under standard conditions. Zebrafish embryos at 48‐hr post‐fertilization (hpf) were dechorinomated prior to cell injection. Cells were fluorescently labeled by 5 μm CM‐DiI (Thermo Fisher Scientific) for 20 min at 37 °C. Approximately 100 CM‐DiI‐labeled cells were injected into embryonic yolk sac using a micropipette and pump (World Precision Instruments). Engrafted embryos were sorted to remove falsely injected embryos and as needed, treated with or without 10 μg·mL^−1^ doxycycline (Sigma‐Aldrich), and maintained at 34 °C for a further 6 days. At 6 days post‐injection (6 dpi), engrafted zebrafish embryos were examined using an Olympus MVX10 Zoom Fluorescence Macro System Microscope. The tumor cells disseminated from the original injection site (yolk sac) to distal regions (head, trunk, and tail) were defined as invasive cells and manually counted. Relative Invasion Index is defined as the number of invasive cells at 6 dpi normalized to the average number of invasive cells in the control group at 6 dpi.

Eight‐week‐old male BALB/c nude mice were obtained from Guangdong Medical Laboratory Animal Center (Guangzhou, China). The mice were housed in a specific pathogen‐free animal facility, and the temperature in the facility was maintained at 22–24 °C with an alternating 12‐h light/dark cycle. OCM1 cells (3*10^6^) with inducible BAP1 knockout and inducible SF3B1 wt/R625H expression were grafted subcutaneously into the right back flank. When tumors were just palpable, mice were fed 0.2 mg·mL^−1^ doxycycline (Sigma‐Aldrich) in drinking water supplemented with 2.5% sucrose over a period of 25 days. Primary tumor masses were then collected and weighted.

### 2.20 Statistical analysis

All data analysis was performed using graphpad Prism version 6.0c for Mac (GraphPad Software, CA). *P* values were noted in the figures. *P* < 0.05 was considered significant.

## 3 Results

### 3.1 Mutual exclusive pattern of *BAP1* and *SF3B1* Mutations in UM patients

**Fig. 1:**
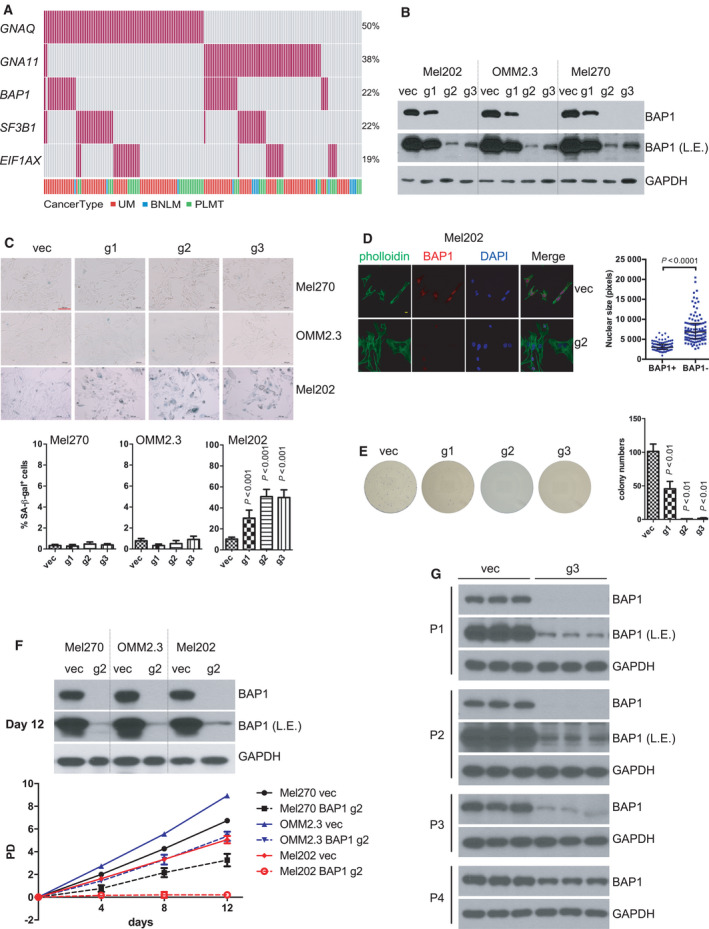
BRCA1‐associated protein 1 (BAP1) deficiency in UM cells with mutant splicing factor 3B subunit 1 (*SF3B1*) induces senescent phenotype. (A) Oncoprint depicting the five genes that are frequently mutated in UM across three studies, including the TCGA uveal melanoma (UM) dataset [4] downloaded from cBioportal [58], the exome sequencing UM dataset [8] and targeted amplicon‐based next‐generation sequencing UM dataset [14], blue nevus‐like melanoma (BNLM) [12, 13] and primary leptomeningeal melanocytic tumor (PLMT) [14]. Each bar in a column represents one patient, and each red bar represents the presence of the specified mutation. Data are from 179 patients. *P* values for mutual exclusivity of the paired gene mutations were derived from Fisher’s exact test. (B) Immunoblots show the knockout (KO) efficiency of three different *BAP1*‐targeting sgRNAs by lentivirus CRISPR‐Cas9. L.E. represents a longer exposure of BAP1 immunoblot. The blots shown are representative of three independent experiments. (C) Images show senescence‐associated β‐galactosidase staining in UM cells after BAP1 KO. G1, g2, g3 represent the three different sgRNAs used in panel B. The scale bar represents 100 μm. Graph represents the percentage of β‐galactosidase‐positive cells versus total cells (at least 200 cells were counted in random fields per group). The data are presented as the mean ± SD (*n* = 3) from one representative experiment out of three. One‐way ANOVA followed by Tukey test was used for statistical analysis. (D) Representative images for nuclear size of Mel202 cells. Immunofluorescence staining was performed for F‐actin (green, phalloidin), BAP1 (red), and DNA (blue, 4’,6‐diamidino‐2‐phenylindole). The scale bar represents 5 μm. Quantification of nuclear size of BAP1‐proficient and BAP1‐deficient Mel202. The data are presented as the median with interquartile range (at least 100 cells were counted in random fields per group) from one representative experiment (*n* = 3) out of three. The Mann–Whitney U‐test was used for statistical analysis. (E) Colony‐formation assay of Mel202 cells infected by vector control (vec) or *BAP1*‐targeting sgRNAs (g1, g2, and g3), and the colonies were stained with crystal violet for quantification. The data are presented as the mean ± SD (*n* = 3) from one representative experiment out of three. One‐way ANOVA followed by Tukey test was used for statistical analysis. (F) Population doublings (PD) of Mel270, OMM2.3, and Mel202 cells infected by vector control (vec) or *BAP1*‐targeting g2 sgRNA followed over 12 days. Immunoblots show the BAP1 protein level to confirm the BAP1 KO efficiency in the respective cell pool at the final time point (12 days). The data are presented as the mean ± SD (*n* = 3) from one representative experiment out of three. (G) Immunoblots show the BAP1 expression recovery during cell passages in Mel202 cells infected by *BAP1*‐targeting g3 sgRNA. The blots shown are representative of three independent experiments.

The top five recurrently mutated genes in UM are *GNAQ*, *GNA11*, *BAP1*, *SF3B1*, and *EIF1AX*. We analyzed available UM samples and observed that *GNAQ* and *GNA11* mutations in 110 of the 118 UM (93.2%) were mutually exclusive (*P* = 1.81e‐20). *BAP1* mutations in 35 UM cases (29.7%) were mutually exclusive with *SF3B1* mutations (25 of 118; 21.2%) (*P* = 0.0066) and *EIF1AX* mutations (15 of 118; 12.7%) (*P* = 0.0051). *SF3B1* and *EIF1AX* mutations tended to be mutually exclusive (*P* = 0.19). Twenty‐five of 35 *BAP1*‐mutant cases were truncating mutations, while most *SF3B1* alterations (20 of 25) encoded an alteration of Arg625. Only two UM harbored both a *SF3B1* and a *BAP1* mutation; the latter included an in‐frame mutation (R610_G619del) and a missense mutation (G185R) (Fig. 1A). Given the fact that other cancer types including BNLM and PLMT share a similar gene mutation profile with UM, we included BNLM and PLMT in our analysis. Across these three cancer types, similar mutually exclusive patterns were found. In this respect, *GNAQ* and *GNA11* mutations were mutually exclusive (*P* = 1.91e‐26); *BAP1* mutations were mutually exclusive with *SF3B1* mutations (*P* = 0.0021) and *EIF1AX* mutations (*P* = 0.00013); *SF3B1* and *EIF1AX* mutations were nearly mutually exclusive (*P* = 0.11) (Fig. 1A and Table S3).

As expected, *BAP1* mRNA expression was significantly (*P* < 0.0001) higher in wild‐type UM cases compared with mutant UM cases, consistent with the observations that most of *BAP1* mutations were truncating mutations (Fig. S1A). However, *SF3B1* gene alterations did not change *SF3B1* mRNA level (Fig. S1B). Of note, *SF3B1*‐mutant UM displayed higher *BAP1* mRNA levels than *SF3B1* wild‐type cases, likely due to the fact that *BAP1* mutations (thus loss of *BAP1* mRNA) occur selectively in the *SF3B1* wild‐type UM tumors (Fig. S1C). Ectopic expression of neither wild‐type nor mutant SF3B1 (R625H) altered BAP1 mRNA and protein levels in Mel270 and OMM2.3 cells, suggesting that SF3B1 does not affect BAP1 expression (Fig. S2).

We investigated the genomic alterations in a panel of 10 cell lines established from primary or metastatic UM by deep mRNA sequencing. Among these UM cell lines, Mel202 contains the heterozygous *SF3B1* mutation (R625G) while the *EIF1AX* mutation (G6D) is detected in 92.1 cells. None of the sequenced UM cell lines harbors *BAP1* mutations (Fig. S3A). Sanger sequencing of genomic DNA from Mel202 and 92.1 further confirmed the above mutations (Fig. S3B). Next, we determined protein expression of BAP1, SF3B1, EIF1AX, and Gq in these UM cell lines. Mesothelioma cell lines NCI‐H2373 and Y‐MESO‐14 served as positive and negative control for BAP1 protein expression, respectively [27]. As shown in Fig. S3C, all UM cell lines showed BAP1, SF3B1, EIF1AX, and Gq protein expression, indicating that mutations in *SF3B1*, *EIF1AX,* or *GNAQ* did not silence their protein expression.

### 3.2 Co‐existing mutations in *BAP1* and *SF3B1* induce senescence

We tested the effect of BAP1 deficiency on UM cell lines with or without SF3B1 mutation. In Mel270 (SF3B1^wt^), OMM2.3 (SF3B1^wt^), and Mel202 (SF3B1^R625G^) cells, we deleted BAP1 by lentivirus CRISPR‐Cas9 system using three different *BAP1*‐targeting single‐guide RNAs (g1, g2, and g3). The knockout efficiency of g2 was highest followed by g3 in the polyclonal population following puromycin selection demonstrated by immunoblotting (Fig. 1B). Upon BAP1 deletion, Mel202 cells grew poorly. We found that BAP1 loss in Mel202 cells displayed senescence‐associated acidic β‐galactosidase (SA‐β‐gal) activity, reminiscent of cellular senescence. Moreover, the frequency of SA‐β‐gal positive cells correlated with BAP1 deletion efficiency in Mel202. Notably, similar deletion of BAP1 did not increase SA‐β‐gal in either OMM2.3 or Mel270 cells, which have wild‐type SF3B1 (Fig. 1C). BAP1‐deficient Mel202 cells also acquired additional senescence phenotypes, including enlarged nuclear size (Fig. 1D), reduction of Lamin B1 (Fig. S4A), and increased senescence‐associated secretary phenotypes (SASP) such as IL‐6 and TNF‐α (Fig. S4B). In contrast, BAP1 loss did not alter these senescent markers in Mel270 or OMM2.3 cells (Figs. S4A–C). These results suggest that the combined mutations in BAP1 (loss of function) and SF3B1 (neomorphic mutation) may cause senescence.

BAP1 deficiency in Mel202 cells strongly inhibited colony formation in a manner proportional to the BAP1 knockout efficiency of the three tested sgRNAs (Fig. 1E). However, the inhibitory effect of BAP1 loss on colony‐forming capacity was substantially less pronounced in Mel270 and OMM2.3 cells than in Mel202 cells (Fig. S4D). Accordingly, the percentage of EdU‐labeled cells (indicative of active DNA synthesis) was significantly decreased in Mel202 BAP1 KO cells but not OMM2.3 BAP1 KO cells (Fig. S4E). BAP1 loss also induced cell cycle G1/G0 phase arrest in Mel202 cells, but not in OMM2.3 cells (Fig. S4F). We next compared the effect of BAP1 deletion on cell growth in UM cell lines. BAP1 loss moderately inhibited the growth of Mel270 and OMM2.3, whereas it completely arrested cell growth in Mel202 cells (Fig. 1F). To further demonstrate that BAP1 deletion inhibits Mel202 growth, we cultured cell pools that were infected with *BAP1* sgRNA g3 which caused an efficient but incomplete deletion of BAP1 (Fig. 1B). Upon successive passages, we observed a gradual increase of BAP1 protein expression, indicating that the BAP1 knockout Mel202 cells were overcompeted during the continuous culture of this heterogeneous cell pool (Fig. 1G). In contrast, we could readily establish BAP1 KO clones from Mel270 and OMM2.3 cells, but not Mel202 cells, after single‐cell sorting (Fig. S5). These data further support a notion that the co‐occurrence of BAP1 deletion and SF3B1 mutation severely compromises cell growth.

**Fig. 2:**
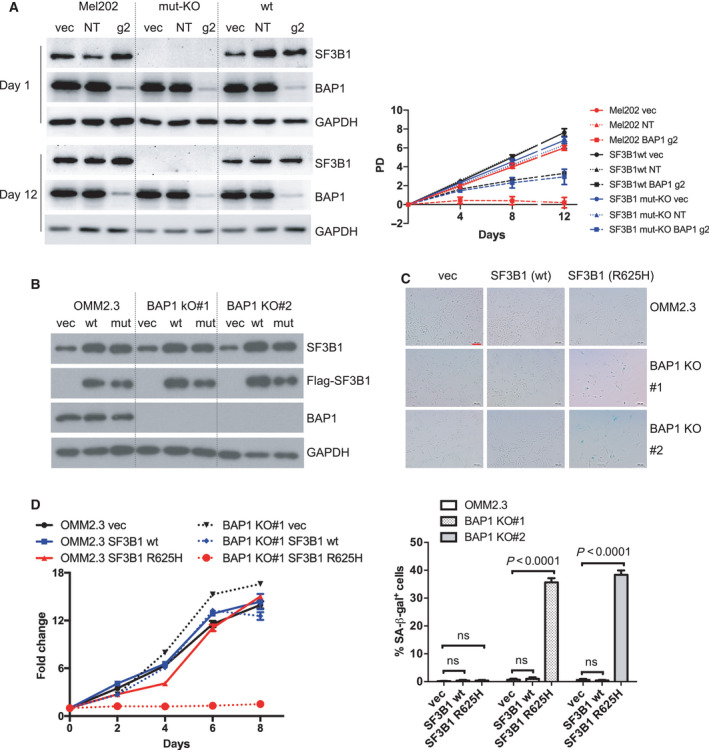
BAP1 deficiency combined with SF3B1 hotspot mutation induces senescence. (A) Growth curve of Mel202 cells with different *SF3B1* mutation status infected by vector control (vec), non‐targeting sgRNA (NT), or *BAP1*‐targeting g2 sgRNA. Mel202 cells harbor heterozygous SF3B1 mutation, SF3B1 mut‐KO Mel202 cells contain out‐frame deletion of the mutant allele and p. His8_Glu9delinsGln in the wild‐type allele, and SF3B1 wt indicates re‐expression of the wild‐type SF3B1 in the SF3B1 mut‐KO cells. The data are presented as the mean ± SD (*n* = 3) from one representative experiment out of three. (B) Re‐expression of Flag‐tagged wt or mutant SF3B1 (R625H) in OMM2.3 and BAP1 KO clones. The blots shown are representative of three independent experiments. (C) Images show acidic senescence‐associated β‐galactosidase staining. The scale bar represents 100 μm. Graph represents the percentage of β‐galactosidase‐positive cells versus total cells (at least 200 cells were counted in random fields per group). The data are presented as the mean ± SD (*n* = 3) from one representative experiment out of three. One‐way ANOVA followed by Tukey test was used for statistical analysis. (D) Growth curve of OMM2.3 (solid lines) and OMM2.3 BAP1 KO#1 clone (dashed lines) infected with retrovirus encoding for wt or mutant SF3B1 (R625H). The data are presented as the mean ± SD (*n* = 6) from one representative experiment out of three.

To further investigate the genetic interaction between *BAP1* and *SF3B1* mutations and to exclude that other alterations in Mel202 might contribute to the incompatibility with BAP1 deletion, we asked whether deletion of the *SF3B1*‐mutant allele in Mel202 cells could reverse BAP1 KO‐induced growth arrest. Mel202 cells have one mutant (R625G) and likely two wild‐type alleles of *SF3B1* [30]. CRISPR‐Cas9 system was used to generate mutant *SF3B1* KO isogenic cell line. After screening a large number of clones, we identified a clone (named as SF3B1 mut‐KO) that has knockout of the mutant allele due to a 1‐bp deletion. This clone also has an in‐frame deletion (3 bp) of the wild‐type alleles, which resulted in the change of His8 to Gln8 and deletion of Glu9 (p. His8_Glu9delinsGln, Figs. S6A–C). We also observed a significant downregulation of *SF3B1* mRNA level in SF3B1 mut‐KO clone by qPCR, which may be due to nonsense‐mediated mRNA decay (NMD) of the mutant transcript with 1‐bp deletion (Fig. S6D). The SF3B1 antibody used in our study recognized the amino terminus of SF3B1 protein; thus, the SF3B1 p. His8_Glu9delinsGln alteration possibly diminished the recognition by the antibody in immunoblotting and immunofluorescence (Figs. 2A and S6E). However, the remaining p. His8_Glu9delinsGln alleles could still contribute to maintaining *in vitro* cell survival in Mel202 SF3B1 mut‐KO clone because it has been demonstrated that survival of Mel202 cells critically depends on the wild‐type copies of *SF3B1* [30]. In order to exclude possible interference induced by SF3B1 p. His8_Glu9delinsGln alteration in Mel202 mut‐KO clone, wild‐type SF3B1 (SF3B1 wt) was stably expressed in the SF3B1 mut‐KO clone, as confirmed by immunofluorescence and western blotting (Figs. S6E and 2A). Importantly, the BAP1 knockout‐induced growth arrest was dramatically rescued when the mutant *SF3B1* allele was deleted in Mel202 cells (Fig. 2A). Together, these observations establish that BAP1 deficiency in addition to SF3B1 mutation causes growth arrest in Mel202.

An important question is whether the senescence phenotype observed in Mel202 is unique or general to other UM cells. Therefore, we sought to determine whether introduction of mutant SF3B1 with BAP1 deficiency could also lead to senescence in another isogenic context. Firstly, we established BAP1 KO clones (BAP1 KO#1 and BAP1 KO#2) from OMM2.3 cells. It should be noted that we could readily obtain BAP1 knockout clones in UM cell lines except Mel202. Immunoblotting (Fig. 2B) and Sanger sequencing (Fig. S5B) confirmed the depletion of protein expression and out‐frame *BAP1* gene editing, respectively. Next, wild‐type and mutant SF3B1 (R625H) were ectopically expressed in the OMM2.3 or BAP1 KO clones (Fig. 2B). Ectopic expression of SF3B1^wt^ did not change SA‐β‐gal staining both in OMM2.3 and BAP1 KO clones. Interestingly, although expression of SF3B1^R625H^ did not alter SA‐β‐gal staining in OMM2.3 cells, it strongly induced accumulation of SA‐β‐gal activity in OMM2.3 BAP1 KO clones (Fig. 2C). Consistently, ectopic expression of SF3B1^wt^ did not change cell growth in OMM2.3 and BAP1 KO#1 clone; however, expression of SF3B1^R625H^ completely blocked the cell growth of BAP1 KO#1 clone while exhibited little effect on OMM2.3 cells (Fig. 2D). These findings show that introduction of mutant SF3B1 induces cellular senescence in BAP1 KO clones but not their isogenic OMM2.3 cells. Collectively, we conclude that co‐existence of BAP1 deficiency and SF3B1 mutation (R625H) induces senescence in UM cells. Our observations thus reveal an explanation for the mutually exclusive nature of *BAP1* and *SF3B1* mutations in UM.

### 3.3 Senescent phenotype induced by BAP1 deficiency and SF3B1 mutation is not dependent on p53

**Fig. 3:**
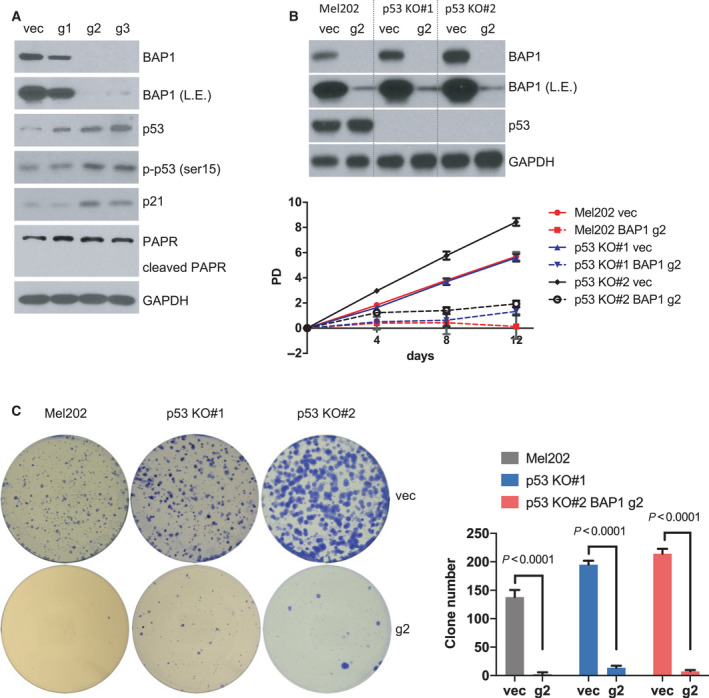
p53 is not required for the senescence phenotype. (A) Immunoblots show the expression of indicated proteins of Mel202 cells infected by vector control (vec) or *BAP1*‐targeting sgRNAs (g1, g2, and g3). The blots shown are representative of three independent experiments. (B) Growth curve of Mel202 and Mel202 p53 KO clones (#1 and #2) infected by vector control (vec) or *BAP1*‐targeting g2 sgRNA. Immunoblots show BAP1 KO efficiency at the final time point (12 days) in Mel202 and Mel202 p53 KO clones (#1 and #2). The data are presented as the mean ± SD (*n* = 3) from one representative experiment out of three. (C) Colony‐formation assay of Mel202 and Mel202 p53 KO clones (#1 and #2) infected by vector control (vec) or *BAP1*‐targeting g2 sgRNA, and the colonies were stained with crystal violet for quantification. The data are presented as the mean ± SD (*n* = 3) from one representative experiment out of three. Student’s *t* test was used for statistical analysis.

The tumor suppressors p53 and p16 INK4A are canonical mediators of cellular senescence [34]. Considering p16 INK4A was undetectable in Mel270, OMM2.3, and Mel202 cells (Fig. S7), we focused on the potential role of p53 in the observed senescence. We observed that BAP1 deficiency in Mel202 cells increased protein expression of p53, phospho‐p53 at ser15 while it did not induce the cleavage of poly (ADP‐ribose) polymerase (PARP, an apoptosis marker) (Fig. 3A). Consistently, expression of p21, which is a p53 target gene involved in cell cycle inhibition and senescence induction, was also induced (Fig. 3A). Since p53 level and its downstream target p21 were elevated by BAP1 deletion in Mel202 cells, we asked whether the senescence phenotype induced by BAP1 and SF3B1 mutations relies on the p53 pathway. We established p53 KO clones (p53 KO#1 and p53 KO#2) from Mel202 cells by CRISPR‐Cas9 system (Fig. S8). Then, BAP1 was deleted in p53‐proficient and p53‐deficient Mel202 cells by CRISPR‐Cas9. Western blotting verified the deletion of p53 protein expression in p53 KO clones and the knockout efficiency of BAP1 in the polyclonal cell pool (Fig. 3B). Deletion of p53 only marginally rescued cell growth arrest (Fig. 3B) as well as the loss of clonogenicity (Fig. 3C) induced by BAP1 deficiency in Mel202 cells.

Although p53 knockout failed to effectively reverse the growth arrest by BAP1 deficiency in Mel202 cells, we noticed there were a few clones formed in p53 KO clones after BAP1 depletion (Fig. 3C). We performed sequential knockout by first infection with *TP53* sgRNA followed by infection with *BAP1* sgRNA; then, cells were sorted into 96‐well plates for single‐cell clones (Fig. S9A). Interestingly, all clones showed no p53 protein expression, suggesting the survival advantages resulted from p53 deletion (Fig. S9B). Of note, we did identify one p53 and BAP1 double knockout clone (dKO #28) (Fig. S9B). However, the dKO clone gradually lost proliferative potential and exhibited a flattened morphology (Fig. S9C), a phenotype reminiscent of senescence. These observations suggest that p53 knockout may provide an initial survival advantage for Mel202 cells upon BAP1 depletion, but does not fully rescue senescence induced by the co‐occurrence of BAP1 deficiency and SF3B1 mutation.

### 3.4 BAP1 deficiency combined with SF3B1 mutation triggers DNA damage response

**Fig. 4:**
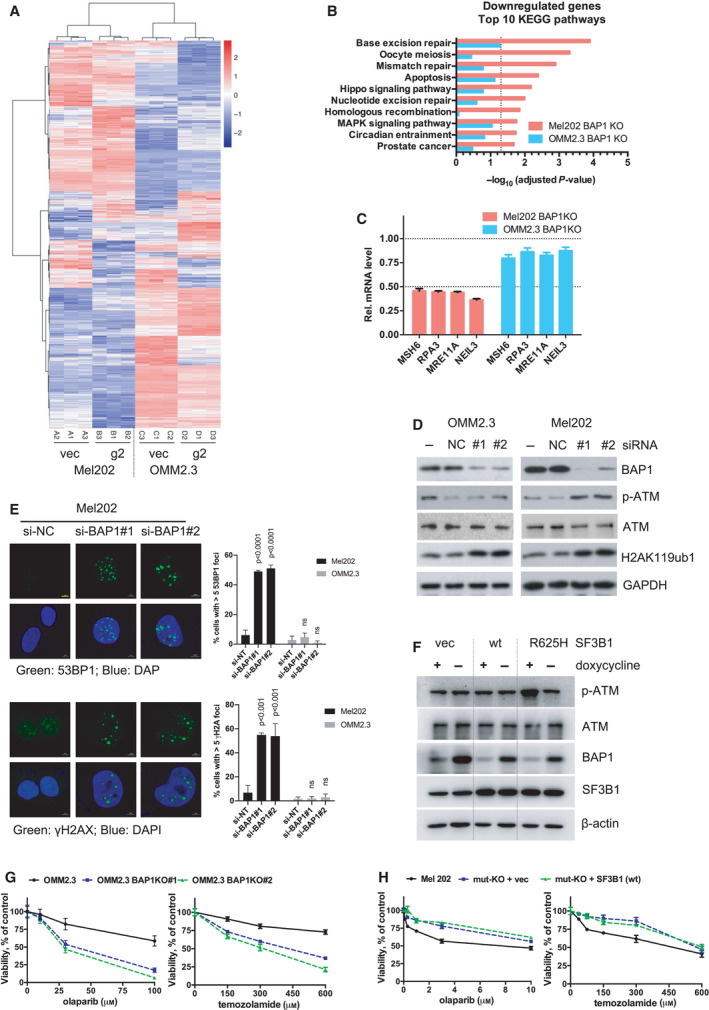
BAP1 deficiency combined with SF3B1 hotspot mutation induces DNA damage response. (A) RNA‐Seq data of differentially expressed genes (DEGs) in Mel202 and OMM2.3 cells with BAP1 KO (g2) compared with vector control (vec). Data were analyzed with DESeq2 (cutoff: adjusted *P*‐value < 0.05). Hierarchical clustering of the DEGs was used to display the log_2_‐fold changes. (B) KEGG pathways enrichment analysis for the downregulated DEGs displayed in the heatmap. Only the top 10 pathways exclusively enriched in Mel202 cells are shown. The bars represent the enrichment scores, ‐log_10_ (adjusted *P*‐value). (C) Expression of DNA‐repair genes was determined by qRT‐PCR after BAP1 KO in Mel202 and OMM2.3 cells. The change of gene expression was normalized to vec control. Error bars represent SD (*n* = 3). (D) Immunoblots show the effect of BAP1 knockdown by siRNA on p‐ATM, total ATM, and H2AK119ub1 in Mel202 and OMM2.3. The blots shown are representative of three independent experiments. (E) Knockdown of BAP1 by siRNA led to increased 53BP1 foci and γH2AX foci in Mel202 but not OMM2.3 cells. The scale bar represents 5 μm. Graphs show mean percentage of cells with > 5 53BP1 or γH2AX foci (more than 100 cells were counted in random fields per group). The data are presented as the mean ± SD (*n* = 3) from one representative experiment out of three. One‐way ANOVA followed by Tukey test was used for statistical analysis. (F) Immunoblots show the effect of inducible BAP1 knockdown on p‐ATM and total ATM in OMM2.3 cells stably expressing vector, wild‐type (wt) SF3B1, or mutant SF3B1 (R625H). Stable OMM2.3 cells expressing inducible *BAP1*‐targeting shRNA were established. These cells were then infected with retroviruses encoding for control vector, wild‐type SF3B1, or mutant SF3B1 (R625H). Cells were treated with doxycycline (200 ng·mL^−1^) for 72 h. The blots shown are representative of three independent experiments. (G) Effects of BAP1 KO on cell sensitivity to DNA damaging agents. Parental OMM2.3 or CRISPR‐mediated BAP1 KO clones (#1 and #2) were treated with olaparib or temozolamide with indicated concentrations for 6 days. Cell viability was determined by MTT assay. The data are presented as the mean ± SD (*n* = 3) from one representative experiment out of three. (H) Effects of SF3B1 mutation status on cell sensitivity to DNA damaging agents. Mel202 cells with different SF3B1 mutation status (heterozygous SF3B1 mutation, SF3B1 mut‐KO, and SF3B1 wt re‐expression in the mut‐KO cells) were treated with olaparib or temozolamide as indicated concentrations for 6 days. Cell viability was determined by MTT assay. The data are presented as the mean ± SD (*n* = 3) from one representative experiment out of three.

To explore molecular mechanism by which BAP1 deficiency combined with SF3B1 mutation induces senescence in UM cells, we performed RNA‐sequencing analysis of Mel202 and OMM2.3 cells with BAP1 deletions mediated by the aforementioned lentiviral CRISPR‐Cas9 system compared with the vector control. Using the resulting mRNA expression profiles, we identified 5216 differentially expressed genes (DEGs, adjusted *P*‐value < 0.05) between wild‐type and BAP1 KO Mel202 cells and 6248 DEGs between wild‐type and BAP1 KO OMM2.3 cells (Fig. S10A and Table S4). A heatmap of the DEGs in these two cell lines upon BAP1 loss was shown in Fig. 4A. To uncover cellular processes that were altered by BAP1 deletion, we preformed enrichment analysis of Kyoto Encyclopedia of Genes and Genomes (KEGG) pathways for DEGs identified in OMM2.3 and Mel202 cells upon loss of BAP1 using KOBAS 3.0 online platform. Downregulated genes in Mel202 cells upon BAP1 deletion are mainly involved in processes related to DNA repair, including base excision repair, mismatch repair, nucleotide excision repair, and homologous recombination. In contrast, these pathways were not significantly enriched by BAP1 deletion in OMM2.3 cells (Fig. 4B and Table S5). Upregulated genes in Mel202 or OMM2.3 upon BAP1 deletion are primarily associated with pathways unrelated to DNA repair (Fig. S10B and Table S5). Quantitative RT‐PCR confirmed that the selected genes related to DNA‐repair pathways were decreased more than 50% in Mel202 cells but only changed slightly in OMM2.3 cells upon BAP1 deletion (Fig. 4C). It is worth noting that DNA damage response defects are intimately linked to cellular senescence. Together, our results suggest that BAP1 loss in cells carrying SF3B1 mutation may impair DNA damage repair capacity and make cells unable to deal with DNA damage stress, thus leading to senescent phenotype.

The enrichment of DNA‐repair pathways in Mel202 cells upon BAP1 deletion promoted us to test whether BAP1 loss in cells with SF3B1 mutation could induce DNA damage response. The mRNA expression of *BAP1* was downregulated by BAP1 siRNA (#1 and #2 target different regions) and the BAP1 knockdown was confirmed by western blotting (Fig. 4D). As expected, BAP1 downregulation was associated with an increase of mono‐ubiquitination of histone H2A at lysine 119 (H2AK119ub1) (Fig. 4D), which is a physiological substrate of BAP1, in both Mel202 and OMM2.3 cells. ATM is a key player in DNA damage response and its activity correlates with phosphorylation. We observed that ATM phosphorylation was increased in Mel202 cells upon siRNA‐mediated BAP1 silencing, whereas knockdown of BAP1 showed no effect on ATM phosphorylation in OMM2.3 cells (Fig. 4D). Moreover, BAP1 loss also led to increased 53BP1‐ and γH2AX‐positive nuclei, indicative of DNA double‐strand breaks, in Mel202 but not OMM2.3 cells (Figs. 4E and S11). These results indicate that BAP1 knockdown preferentially induced genomic stress in the SF3B1‐mutant Mel202 cells.

We made an inducible BAP1 shRNA (Tet‐pLKO‐puro system) and successfully established OMM2.3 stable cells. Next, wild‐type or mutant SF3B1 (R625H) was ectopically expressed to a level comparable with endogenous SF3B1 in OMM2.3 stable cells, in which BAP1 expression could be effectively reduced upon BAP1 shRNA induction by doxycycline treatment (Fig. 4F). We examined ATM phosphorylation as a marker for DNA damage stress. Notably, ATM phosphorylation was increased only in OMM2.3 cells that have both BAP1 knockdown and mutant SF3B1 expression. No elevation of ATM phosphorylation was observed in either BAP1 knockdown or mutant SF3B1 expression (Fig. 4F). Therefore, the co‐occurrence of BAP1 knockdown and SF3B1 mutation induces DNA damage response.

To assess the role of BAP1 and SF3B1 in DNA damage repair, we examined cellular sensitivity to drugs that inhibit DNA repair or induce DNA damage. PARP is an important player in DNA damage repair. We observed that BAP1 deletion sensitized OMM2.3 cells to PARP inhibitor olaparib and chemotherapeutic drug temozolamide (Fig. 4G). Similarly, BAP1 KO OCM1 cells were also more sensitive to these two agents than the control cells (Fig. S12). Moreover, deletion of the mutant SF3B1 significantly increased Mel202 cell resistance to the PARP inhibitor and temozolamide though the effect was moderate (Fig. 4H).

Splicing factor mutations have been demonstrated to cause accumulation of DNA–RNA hybrids (also known as R‐loops), leading to replication stress [35]. Specific knockout of the mutant *SF3B1* allele decreased R‐loops, evidenced by reduced phosphorylation of single‐strand binding protein RPA32 at ser33 (Fig. S13A) and weakened S9.6 staining (Fig. S13B). Importantly, ectopic expression of RNasH1, which specifically degrades RNA‐DNA hybrids, decreased S9.6 staining and p‐RPA (ser33) in Mel202 cells, indicating that the observed immunofluorescence signals were from the binding of S9.6 to R‐loops (Figs. S13C and D). An interesting question arising from our data was whether R‐loops contribute to the growth arrest induced by BAP1 and SF3B1 mutations. In this respect, neither ectopic expression of BAP1 nor knockdown of BAP1 influenced R‐loop formation (Figs. S14A and B). Moreover, alleviation of R‐loops by ectopic RNaseH1 expression failed to rescue BAP1 loss‐induced growth arrest in Mel202 cells (Fig. S14C). These data suggest that the generation of R‐loops by mutant SF3B1 is not directly linked to cell growth arrest induced. Consistently, ectopic expression of RNaseH1 did not mitigate BAP1 loss‐induced 53BP1 and γH2AX foci formation, suggesting that the observed DNA damage occurs independently of R‐loops (Fig. S15A and B).

On the basis of the above data, we speculate that either BAP1 deletion or SF3B1 mutation makes cells more vulnerable to DNA damage. UM cells can survive either BAP1 loss or SF3B1 mutation alone; however, they cannot tolerate the co‐occurrence of BAP1 loss and SF3B1 mutation due to impairment of DNA‐repair capacity, eventually leading to senescent phenotype and growth arrest.

### 3.5 BAP1 and SF3B1 double mutation inhibits invasion of UM cells *in vivo*

**Fig. 5:**
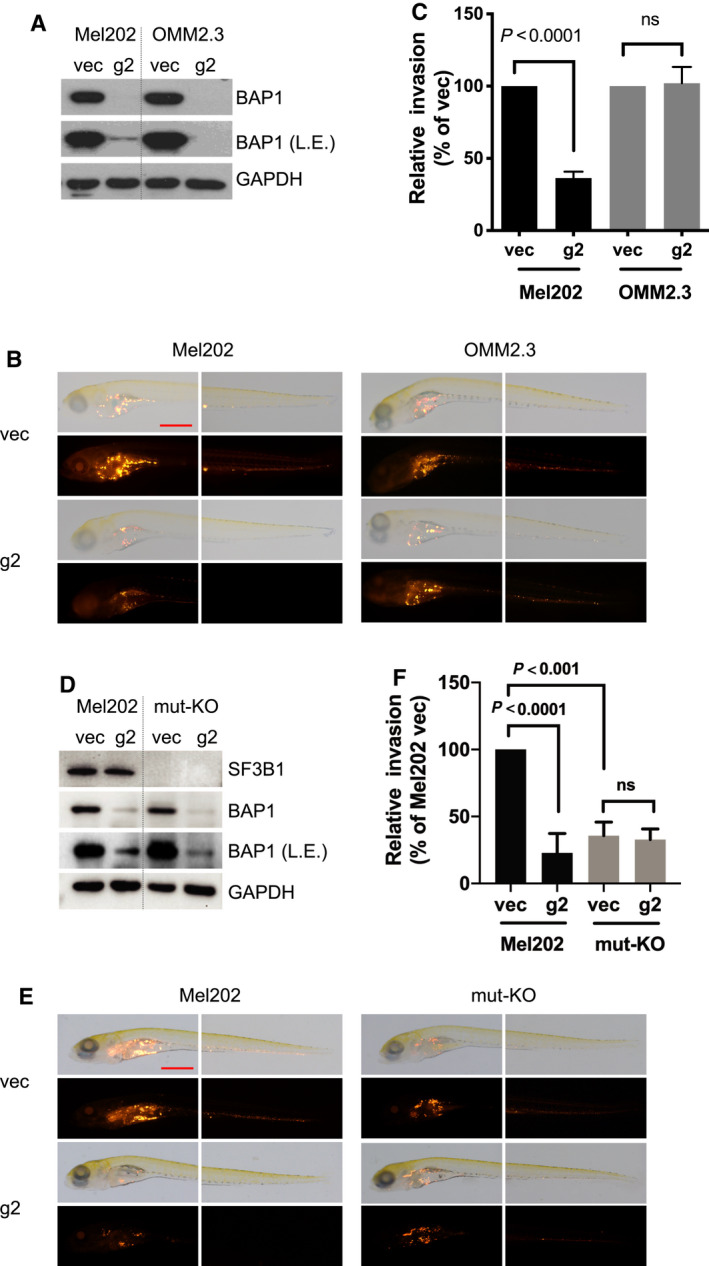
BAP1 deletion suppresses *in vivo* invasion of SF3B1‐mutated UM cells. (A) Immunoblots show BAP1 KO efficiency by lentivirus CRISPR‐Cas9. The blots shown are representative of three independent experiments. (B) Representative images at 6 days post‐injection (dpi) of vector and CRISPR‐mediated BAP1 KO Mel202/OMM2.3 heterogeneous cell pool engrafted into the yolk sac of zebrafish embryos. The images shown are representative of three independent experiments. The scale bar represents 500 μm. (C) Graphs show quantification of tumor cell invasion. All experiments consist of three independent repeats. For each independent experiment, at least 10 embryos were used in each group. Values of three independent experiments were given as mean ± SD. Student’s *t* test was used for statistical analysis. (D) Immunoblots show BAP1 KO efficiency by lentivirus CRISPR‐Cas9. The blots shown are representative of three independent experiments. (E) Representative images at 6 dpi of vector and CRISPR‐mediated BAP1 KO Mel202/Mel202 SF3B1 mut‐KO heterogeneous cell pool engrafted into the yolk sac of zebrafish embryos. The images shown are representative of three independent experiments. The scale bar represents 500 μm. (F) Graphs show quantification of tumor cell invasion. All experiments consist of three independent repeats. For each independent experiment, at least 10 embryos were used in each group. Values of three independent experiments were given as mean ± SD. Student’s *t* test was used for statistical analysis.

To test whether BAP1 deletion affects cell migration of SF3B1‐mutant UM cells, we first performed transwell assays *in vitro*. BAP1 deletion significantly decreased migration capacity of Mel202 cells but not OMM2.3 cells (Fig. S16A). We next investigated the effect of BAP1 deficiency in addition to SF3B1 mutation on UM cancer cell migration in zebrafish xenograft models. The transparent and immune‐privilege nature of zebrafish embryos allows direct visualization of single human tumor cell migration, metastatic intravasation, and extravasation *in vivo*. It has been demonstrated that UM cells derived from metastasis showed an increased ability to disseminate away from the yolk (implantation site) than UM cells derived from the primary UM tumors [36]. BAP1 was deleted by the aforementioned lentiviral CRISPR‐Cas9 system (Fig. 5A). BAP1 knockout polyclonal cell pool and empty vector‐transduced control cell pool were fluorescently labeled by CM‐DiI before they were injected into the yolk sac of 2‐day‐old zebrafish embryos. Tumor cell invasion in the zebrafish body was monitored at six days after injection (6 dpi). Dissemination of tumor cells from the original injection site (yolk sac) to distal regions (head, trunk and tail) was observed. There was no obvious change in tumor cells disseminated away from yolk sac in vector and BAP1 KO groups of OMM2.3 cells. However, compared with vector control group, there were significantly fewer tumor cells disseminated away from the yolk sac in the Mel202 BAP1 KO cells, indicating the inhibitory effect of BAP1 deletion on invasion in Mel202 cells, which have SF3B1 mutation, but not OMM2.3 cells (Figs. 5B and C). Mel202 SF3B1 mut‐KO clone showed a significant decreased ability of dissemination compared to its isogenic control Mel202 cells, which is consistent with the pro‐metastatic effect of SF3B1 oncogenic mutations (Figs. 5D–F). Alleviation of R‐loops by ectopic expression of RNaseH1 in Mel202 cells did not mimic the inhibitory effect of mutant SF3B1 deletion on cell migration (Figs. S16B and C). Of note, BAP1 deletion failed to further decrease tumor cell dissemination in Mel202 SF3B1 mut‐KO clone (Figs. 5D–F), suggesting the inhibitory effect of the co‐occurrence of BAP1 deletion and SF3B1 mutation on invasion.

**Fig. 6:**
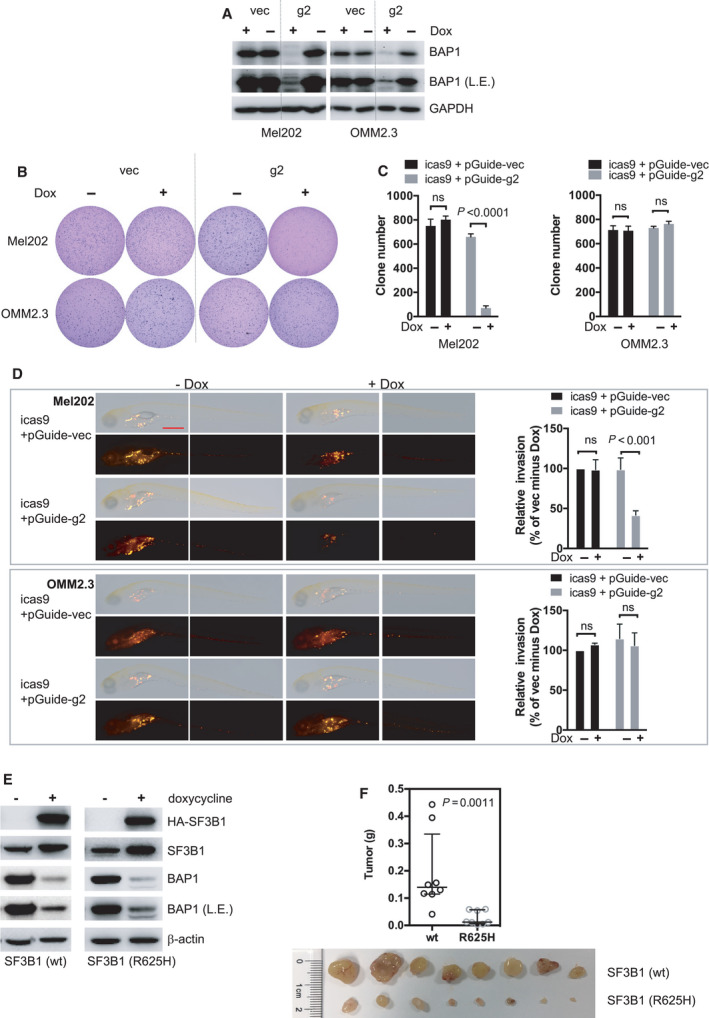
BAP1 deficiency combined with SF3B1 mutation suppresses *in vivo* invasion and growth of UM cells. (A) Immunoblots show BAP1 KO efficacy by inducible lentivirus CRIPSR‐Cas9. Stable Mel202 and OMM2.3 cell clones with Cas9 expression under doxycycline‐inducible promoter were selected. These Cas9‐expressing clones were then infected with lentivirus encoding for control vector (vec) or *BAP1*‐targeting sgRNA (g2). Cells were treated with doxycycline (200 ng·mL^−1^) for 6 days. The blots shown are representative of three independent experiments. (B) Soft‐agar colony‐formation assay of Mel202 and OMM2.3 cells upon inducible BAP1 deletion. Colonies were stained with crystal violet. The images shown are representative of three independent experiments. (C) Quantification of colonies. The data are presented as the mean ± SD (*n* = 3) from one representative experiment out of three. Student’s *t* test was used for statistical analysis. (D) Representative images at 6 dpi of parental and inducible CRISPR‐mediated BAP1 knockout Mel202 and OMM2.3 heterogeneous cell pool engrafted into the yolk sac of zebrafish embryos. The scale bar represents 500 μm. After injection, embryos were soaked in egg water containing doxycycline (10 μg·mL^−1^). Graphs show quantification of tumor cell invasion. All experiments consist of three independent repeats. For each independent experiment, at least 10 embryos were used in each group. Values of three independent experiments were given as mean ± SD. Student’s *t* test was used for statistical analysis. (E) Immunoblots show inducible BAP1 knockout and inducible expression of wild‐type or mutant SF3B1 (R625H) in OCM1 cells. Cells were treated with doxycycline (200 ng·mL^−1^) for 3 days, and BAP1 deletion and wild‐type/mutant SF3B1 were assessed by immunoblotting. The blots shown are representative of three independent experiments. (F) BAP1 knockout combined with mutant SF3B1 expression inhibits OCM1 tumor xenograft growth *in vivo*. The same cell lines used in (E) were grafted into nude mice subcutaneously, and tumor weight was quantified. Data are presented as median with interquartile range. *n* = 8 mice per group. The Mann–Whitney U‐test was used for statistical analysis.

Next, we investigated tumor cell migration in an inducible BAP1 KO model in which BAP1 was deleted after cells were injected into yolk sac. We made a doxycycline‐inducible Flag‐Cas9 expression by transducing cells with pCW‐Cas9 (Tet‐on promoter) and selected Mel202 and OMM2.3 clones with inducible Cas9 expression (Figs. S17A and B). These clones were then transduced with pGuide‐hygro lentiviral construct expressing *BAP1*‐targeting sgRNA (g2). We confirmed that BAP1 expression in these clones was effectively reduced upon doxycycline treatment, which induced Flag‐Cas9 expression (Fig. 6A). Doxycycline treatment significantly decreased anchorage‐independent growth of SF3B1‐mutant Mel202 cells but not SF3B1 wild‐type OMM2.3 cells *in vitro* (Figs. 6B and C). Moreover, doxycycline‐induced BAP1 deletion greatly suppressed dissemination of Mel202 cells but not OMM2.3 cells in zebrafish (Fig. 6D). Collectively, these observations suggest that BAP1 deletion preferentially suppresses tumor cell invasion of SF3B1‐mutant Mel202 cells, but not the SF3B1 wild‐type OMM2.3 cells, in xenograft zebrafish models. The observed suppression of invasion could be due to direct effects of BAP1 loss on migration of SF3B1‐mutant cells as well as indirect effects of senescence‐related changes in cell proliferation and motility.

### 3.6 Constitutive Gq mutation is a UM vulnerability but not required for growth inhibition induced by BAP1 and SF3B1 double mutations

To investigate whether the senescent phenotype induced by BAP1 deficiency and SF3B1 mutation is dependent on the constitutively active Gq signaling as most UM have activated mutations in this pathway, we attempted to delete Gq in Mel202 and OMM2.3. Mel202 has one wild‐type and one mutant (Q209L; R210K) *GNAQ* allele (Fig. S18A) whereas OMM2.3 has two homozygous mutant alleles (Q209L) (Fig. S18B). Gq protein could be effectively reduced by transient infection with each of the four different sgRNAs (Fig. S18C). We found that much fewer colonies could be obtained from Mel202 cells after single‐cell sorting when compared to similar experiments for deletion of p53 or SF3B1. We were unable to obtain clones with functional inactivation of the mutant *GNAQ* allele. Interestingly, among the six clones sequenced, there were five out‐frame indels and one in‐frame indels in the wild‐type allele. For the mutant allele, however, five clones had in‐frame indels (3, 6, 9 bp indels) and one had a missense mutation (Q176K) (Fig. S18D). These results indicate that the mutant GNAQ allele cannot be inactivated in Mel202. For the OMM2.3 cells, there was no difficulty to obtain single‐cell clones. We sequenced 32 clones that had reduced Gq protein levels based on western blotting. However, we failed to obtain any clone that had out‐frame editing in both alleles. Instead, we found that all 32 clones had in‐frame editing in at least one *GNAQ* allele, confirming the high efficiency of the CRISPR‐Cas9 technology in our experiments. Among the nine clones having genomic editing in both alleles, all had one allele with 6 bp in‐frame indels while the other allele was out‐frame indel in eight out of the nine clones. Among the 23 clones having single *GNAQ* allele editing, 19 clones had out‐frame indels, consistent with their lower Gq protein level based on our initial western blotting screen (Fig. S18E). Collectively, these data provide compelling evidence that the mutant Gq is essential for cell growth and/or survival of UM harboring the mutation. Our data suggest that targeting active Gq/11 or their signaling will be an attractive approach to treat UM as more than 90% UM cancers have activating mutation in Gq/11.

Finally, we tested the OCM1 which does not have *GNAQ/11* mutation, but instead has an active *BRAF* V600E mutation (Fig. S3A). We first established doxycycline‐inducible BAP1 KO OCM1 cells by the aforementioned lentiviral Tet‐on CRISPR‐Cas9 system (Figs. S17C and D). Next, doxycycline‐inducible wild‐type or mutant SF3B1 was introduced by pRetroX‐Tight‐GFP retroviral expression construct. In this system, BAP1 deletion and ectopic SF3B1 overexpression were achieved simultaneously upon doxycycline treatment (Fig. 6E). Similar to data observed in the Gq mutant UM cells, ectopic expression of mutant SF3B1, but not the wild‐type SF3B1, combined with BAP1 deletion strongly impaired tumor growth in a nude mouse xenograft model (Fig. 6F). Together, these observations suggest that co‐induction of BAP1 deficiency and SF3B1 mutation suppresses melanoma cell growth independent of the *GNAQ/11* mutation status.

## 4 Discussion

**Fig. 7:**
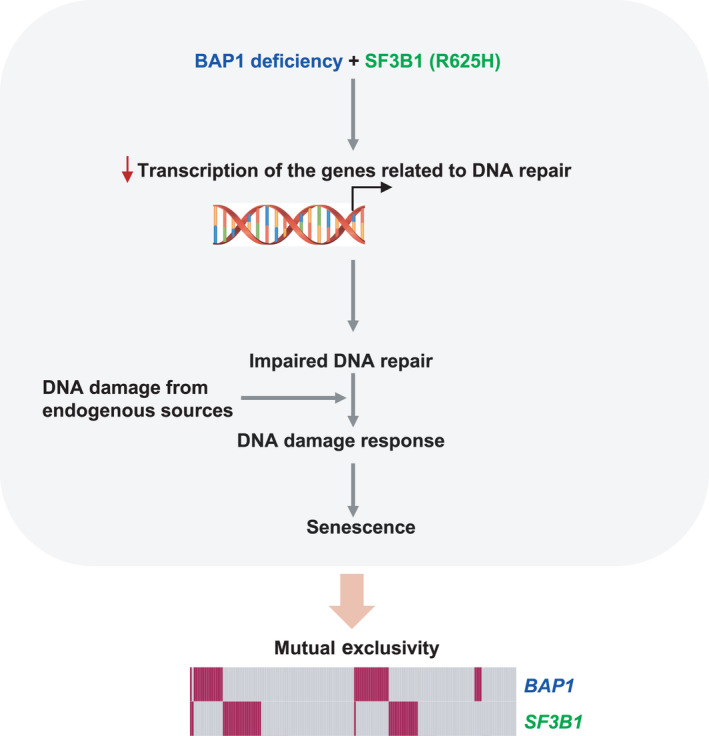
A proposed model accounting for the observed mutually exclusive pattern of *BAP1* and *SF3B1* mutations in UM. The transcriptional suppression of DNA‐repair genes derived from co‐occurrence of BAP1 deficiency and SF3B1 hotspot mutation (R625H) impairs cells’ capacity to buffer endogenous DNA damage and consequently leads to DNA damage and senescence. These data provide a functional explanation for the observed mutual exclusivity of *BAP1* and *SF3B1* mutations in UM.

Recurrent mutations in *BAP1* and *SF3B1* are correlated with high and intermediate risk of metastasis in UM patients, respectively. Here, we identify that co‐occurrence of BAP1 deficiency and SF3B1 hotspot mutation leads to cellular senescence due to compromised DNA damage response (Fig. 7).

DNA damage, occurring as a result of endogenous metabolic reaction and replication stress or from exogenous sources like radiation and chemotherapeutics, is a common stress which can trigger cellular senescence. Both BAP1 and SF3B1 have been described to participate in DNA repair. BAP1 deficiency impairs homologous recombination, which might make cells more reliant on other DNA‐repair pathways and confer a higher sensitivity to PARP inhibition [37]. SF3B1 has been described to be involved in DNA repair in hematological tumors as well [38]. In agreement with these findings, our study shows that UM cells with BAP1 deficiency or SF3B1 mutation are more sensitive to PARP inhibitor and temozolamide compared to their wild‐type counterparts, suggesting compromised DNA‐repair capacity induced by BAP1 loss or SF3B1 mutation. This observation implies a therapeutic potential of DNA damaging agents for cancers with *BAP1* or *SF3B1* mutations. Our RNA‐Seq‐based transcriptome analysis shows BAP1 deletion in SF3B1‐mutant UM cells but not SF3B1 wild‐type cells downregulate genes that are enriched in multiple DNA‐repair pathways. It is thus tempting to speculate that the transcriptional suppression of DNA‐repair genes derived from co‐occurrence of BAP1 deletion and SF3B1 mutation impairs cells’ capacity to buffer DNA damage from endogenous sources and consequently leads to DNA damage and senescence. A recent study connecting H2A deubiquitinating activity of BAP1 to DNA replication fork progression shows that the dynamic balance of ubiquitination and deubiquitination of H2A can prevent the formation of R‐loops and thereby the occurrence of replication stress [39]. Our study also shows that BAP1 deletion maintained H2A of UM cells at a hyperubiquitination status while selective knockout of the *SF3B1*‐mutant allele in Mel202 cells decreased R‐loops. However, modification of BAP1 expression does not influence R‐loops. More importantly, alleviation of R‐loops by ectopic expression of RNaseH1 fails to rescue growth arrest and DNA damage induced by BAP1 deletion in SF3B1‐mutant cells. Based on our data, the observed senescent phenotype is unlikely associated with R‐loops. Of note, alleviation of R‐loops also does not attenuate migration of SF3B1‐mutant cells, suggesting that other aberrations induced by SF3B1 mutations might be responsible for reduced cell migration. Future studies are needed to address molecular mechanisms by which senescence and reduced migration induced by BAP1 loss in SF3B1 mutant contribute to invasion capacity in UM tumors.

Gq/11 knockdown inhibits the growth of Gq/11‐mutated UM cell lines, whereas such effect was not observed in Gq/11 wild‐type cell lines [40], indicating that Gq/11‐mutated UM is Gq/11 addictive. Consistently, we sequenced a large number of single‐cell clones derived from CRISPR‐Cas9‐treated Mel202 (heterozygous *GNAQ* mutation) or OMM2.3 (homozygous *GNAQ* mutation) and found all clones remain at least one functional allele of the mutant *GNAQ* with in‐frame editing or no editing. Similar to *GNAQ/11*, *SF3B1* functions as an oncogene whose hotspot mutations confer a change of function. However, single‐cell clones derived from CRISPR‐Cas9‐mediated *SF3B1* gene editing in Mel202 cells (heterozygous SF3B1 mutation) retain at least one functional wild‐type allele with in‐frame editing, which is concordant with previous findings that cancer cells bearing *SF3B1* mutations exhibit dependency on the remaining wild‐type *SF3B1* allele, but not the mutant allele, for survival [30, 41]. Thus, although selective deletion of mutant SF3B1 has been reported to reverse aberrant splicing, it failed to translate to growth‐inhibitory effects on SF3B1‐mutated cancer cell lines [30]. These findings raise concerns as to whether specific targeting of SF3B1‐mutant protein will yield robust antitumor effect. Moreover, SF3B1 appears to be essential in normal cells without SF3B1 alterations [30]. The fact that current SF3B1 small‐molecule inhibitors bind and inhibit both wild‐type and mutant SF3B1 raises potential cautions regarding the therapeutic index for SF3B1 inhibitors.

Classification of UMs has been based on chromosome copy number changes [42], multi‐gene expression profile (GEP) [43], and integrative analysis [4]. Prognostically favorable class 1 UMs are featured by disomy 3, class 1 GEP, and mutation in *EIF1AX* or *SF3B1*. By contrast, prognostically poor class 2 UMs show monosomy 3, class 2 GEP, and mutation in *BAP1* [25]. With regarding of UM tumorigenesis, almost all UM tumors carry a mutation in Gq signaling (due to mutations in *GNAQ*, *GNA11*, *PLCB4*, or *CYSLTR2*). These primary mutation events contribute to tumor initiation while subsequence mutations in other genes, such as *BAP1*, *SF3B1*, and *EIF1AX*, contribute to tumor progression and metastasis [25]. UM tumors are common in cell type origin (ocular melanocyte) and primary genetic alteration (Gq signaling). Interestingly, BAP1 depletion by siRNA in UM cell lines *in vitro* shifted the GEP toward the class 2 signature [9]. The existence of rare *BAP1* mutations in Class 1 UM tumors also suggests that *BAP1* mutations may precede the emergence of the class 2 signature [9]. These findings indicate that BAP1 mutations may contribute to the progression from class 1 to class 2. Naturally, genetic mutations will affect tumor property, thus tumor classification. One could argue that *BAP1*‐mutant tumors and *SF3B1*‐mutant tumors are in different subclasses. However, using this argument to explain the mutual exclusivity can be circular. Here, we present a more informative explanation for the mutual exclusivity based on the growth defect caused by the combined mutations of both *BAP1* and *SF3B1* from isogenic cell pairs.

Synthetic lethality/sickness is an interaction between two genetic events whereby either genetic event alone has little effect on cell viability but the co‐occurrence of both events leads to cell death/unfitness [44]. The presence of one of these genetic perturbations in cancer cells but not in normal cells can therefore create opportunities for targeted therapy for defined patient populations. Recently, a number of studies have carried out large‐scale synthetic lethality screening in cell culture using CRISPR‐Cas9‐mediated knockout or RNA interference. These unbiased screens map a genome‐wide synthetic lethality network [45, 46, 47, 48]. Nonetheless, it is unpredictable whether the observed synthetic lethal interactions derived from cell‐based screening are translatable *in vivo*. To overcome this limitation, inferring synthetic lethal interactions from mutually exclusivity of genetic events observed in actual cancer patients serves as a complementary strategy. In some cases, genes mutated in a mutually exclusive pattern within a tumor type have been found to be players in the same functional pathways, which can be explained by functional redundancy. The observed mutual exclusivity of *GNAQ* and *GNA11* mutations in UM falls into this category. In contrast, some mutually exclusive aberrations in the same tumor types could be due to synthetic unfitness where simultaneous mutations in both genes are detrimental for cancer progression [44]. In such a case, synthetic lethality of paired mutations might provide cancer vulnerability for targeted therapy. In this study, we show that mutual exclusivity of *BAP1* and *SF3B1* mutations in UM patients is due to senescence phenotype induced by co‐occurrence of these two mutations.

We noticed the co‐existence of mutations in *SF3B1* and *BAP1* in two out of 118 UM cases. This appears to be inconsistent with our model that *SF3B1* mutation combined with *BAP1* deletion induces cellular senescence. Notably, we have identified one *BAP1* and *TP53* double knockout clone from *SF3B1*‐mutant Mel202 cells by single‐cell sorting, suggesting that p53 deficiency can rescue the senescence induced by mutations in both *BAP1* and *SF3B1*, at least in our experimental setting. Thus, it is possible that other genetic or epigenetic alterations might help cells bypass senescence associated with the combined mutations of *BAP1* and *SF3B1*, leading to the co‐existing mutations of these two genes in rare tumor cases. Indeed, many synthetic lethal interactions are likely to be context dependent. Various factors, such as background mutations, can affect synthetic lethal interactions [49, 50]. For example, loss of 53BP1 or components of the non‐homologous end‐joining pathway can suppress the synthetic lethal interaction between poly (ADP‐ribose) polymerase 1 (PARP1) and the breast cancer susceptibility gene *BRCA1* [44]. It is worth mentioning that statistically significant mutual exclusivity indicates a negative association between two genomic alterations rather than the absolute absence of co‐occurrence of both alterations. For example, there are rare tumors carrying both mutations in multiple mutual exclusive gene pairs that display synthetic lethality [51, 52, 53, 54, 55, 56]. The existence of rare tumors carrying double mutations in a synthetic lethal gene pair does not nullify the synthetic lethal concept, but instead reflects the complexity of the cancer (such as genetic/epigenetic backgrounds and microenvironment) which contributes to drug resistance to synthetic lethal therapy in clinical practice [57].

## 5 Conclusions

Together, our data show an intolerability of combined mutations of both *BAP1* and *SF3B1*, providing a mechanistic explanation for the observed mutual exclusivity of these two genes in UMs. We further speculate that this intolerability may also be applicable to blue nevus‐like melanoma and primary leptomeningeal melanocytic tumor as they display genetic mutations essentially identical to UM.

## Conflict of interest

KLG is a cofounder of and has equity interest in Vivace Therapeutics. The terms of this arrangement have been reviewed and approved by the University of California San Diego in accordance with its conflict of interest policies. The other authors declare that they have no conflict of interest.

### Peer review

The peer review history for this article is available at https://publons.com/publon/10.1002/1878‐0261.13128.

## Author contributions

KLG, LY, and SL involved in conception and design. LY, DZ, GZ, ZR, XL, PL, SWP, ZM, TM, YL, and YZ contributed to development of methodology. LY, DZ, GZ, and XL acquired data. LY, DZ, GZ, ZR, XL, and PL analyzed and interpreted the data. KLG and LY involved in writing, review, and revision of the manuscript. LY, SWP, ZM, TM, YL, YZ, SL, JHB, and KLG contributed to administrative, technical, or material support. KLG, LY, and SL supervised the study.

## Supporting information


**Fig. S1**. mRNA expression of BRCA1‐associated protein 1 (*BAP1*) and splicing factor 3B subunit 1 (*SF3B1*) in TCGA uveal melanoma (UM) dataset.

**Fig S2**. Ectopic expression of either wild‐type or mutant SF3B1 did not alter BAP1 expression in Mel270 and OMM2.3 cells.

**Fig S3**. Genomic mutations and protein expression of *GNAQ*, *GNA11*, *BRAF*, *BAP1*, *SF3B1* and *EIF1AX* in multiple UM cell lines.

**Fig S4**. BAP1 deficiency in UM cells with mutant *SF3B1* induces senescent phenotype.

**Fig S5**. Identification of BAP1 KO clones from Mel270 and OMM2.3 cells.

**Fig S6**. Identification of Mel202 SF3B1 mut‐KO clone.

**Fig S7**. The expression of p16 INK4A is absent in Mel270, OMM2.3 and Mel202 cells.

**Fig S8**. Sanger DNA sequencing shows genomic editing of *TP53* introduced by CRISPR‐Cas9 in Mel202 p53 KO clones (#1 and #2).

**Fig S9**. Identification and characterization of *BAP1* and *TP53* double knockout clone from Mel202 cells.

**Fig S10**. KEGG pathways enrichment analysis for the identified DEGs.

**Fig S11**. BAP1 knockdown does not cause DNA damage in OMM2.3 cells.

**Fig S12**. Effects of BAP1 KO on cell sensitivity to DNA damaging agents in OCM1 cells.

**Fig S13**. Deletion of mutant SF3B1 in Mel202 cells alleviates R‐loop formation.

**Fig S14**. Alleviation of R‐loops does not rescue BAP1 deletion‐induced growth arrest in Mel202 cells.

**Fig S15**. Alleviation of R‐loops does not attenuate BAP1 loss‐induced DNA damage in Mel202 cells.

**Fig S16**. BAP1 deletion decreases cell migration in Mel202 but not OMM2.3 cells.

**Fig S17**. Screening doxycycline‐inducible BAP1 KO clones.

**Fig S18**. Characterization of genomic editing of *GNAQ* by CRISPR‐Cas9.

**Table S1**. Guide sequences used.

**Table S2**. Antibodies used.

**Table S3**. Mutation status of *GNAQ*, *GNA11*, *BAP1*, *SF3B1* and *EIF1AX* in uveal melanoma (UM), blue nevus‐like melanoma (BNLM) and primary leptomeningeal melanocytic tumor (PLMT) cases.

**Table S4**. Lists of differentially expressed genes (DEGs).

**Table S5**. KEGG pathways enrichment analysis for the identified DEGs.

## Data Availability

The RNA‐seq data have been deposited to National Center for Biotechnology Information (NCBI) via the Sequence Read Archive (SRA) database repository with the dataset identifier (Study SRA BioProject accession number NO.: PRJNA596363). All other data that support the findings of this study are available upon request.

## References

[1] Arnesen K (1985) The neural crest origin of uveal melanomas. Int Ophthalmol 7, 143–147. 3997355 10.1007/BF00128360

[2] Singh AD , Bergman L & Seregard S . (2005) Uveal Melanoma: epidemiologic aspects. Ophthalmol Clin N Am 18, 75–84. 10.1016/j.ohc.2004.07.002 15763193

[3] Diener‐West M , Reynolds SM , Agugliaro DJ , Caldwell R , Cumming K , Earle JD , Hawkins BS , Hayman JA , Jaiyesimi I , Jampol LM *et al*. (2005) Development of metastatic disease after enrollment in the COMS trials for treatment of choroidal melanoma: Collaborative Ocular Melanoma Study Group Report No. 26. Arch Ophthalmol 123, 1639–1643. 16344433 10.1001/archopht.123.12.1639

[4] Robertson AG , Shih J , Yau C , Gibb EA , Oba J , Mungall KL , Hess JM , Uzunangelov V , Walter V , Danilova L *et al*. (2017) Integrative analysis identifies four molecular and clinical subsets in uveal melanoma. Cancer Cell 32, 204–220. 28810145 10.1016/j.ccell.2017.07.003 PMC5619925

[5] Yu FX , Luo J , Mo JS , Liu G , Kim YC , Meng Z , Zhao L , Peyman G , Ouyang H , Jiang W *et al*. (2014) Mutant Gq/11 promote uveal melanoma tumorigenesis by activating YAP. Cancer Cell 25, 822–830. 24882516 10.1016/j.ccr.2014.04.017 PMC4075337

[6] Henriquez F , Janssen C , Kemp EG & Roberts F (2007) The T1799A BRAF mutation is present in iris melanoma. Invest Ophthalmol vis Sci 48, 4897–4900. 17962436 10.1167/iovs.07-0440

[7] Harbour JW , Roberson ED , Anbunathan H , Onken MD , Worley LA & Bowcock AM (2013) Recurrent mutations at codon 625 of the splicing factor SF3B1 in uveal melanoma. Nat Genet 45, 133–135. 23313955 10.1038/ng.2523 PMC3789378

[8] Martin M , Masshofer L , Temming P , Rahmann S , Metz C , Bornfeld N , van de Nes J , Klein‐Hitpass L , Hinnebusch AG , Horsthemke B *et al*. (2013) Exome sequencing identifies recurrent somatic mutations in EIF1AX and SF3B1 in uveal melanoma with disomy 3. Nat Genet 45, 933–936. 23793026 10.1038/ng.2674 PMC4307600

[9] Harbour JW , Onken MD , Roberson ED , Duan S , Cao L , Worley LA , Council ML , Matatall KA , Helms C & Bowcock AM (2010) Frequent mutation of BAP1 in metastasizing uveal melanomas. Science 330, 1410–1413. 21051595 10.1126/science.1194472 PMC3087380

[10] Karlsson J , Nilsson LM , Mitra S , Alsen S , Shelke GV , Sah VR , Forsberg EMV , Stierner U , All‐Eriksson C , Einarsdottir B *et al*. (1894) Olofsson Bagge R & Nilsson JA (2020) Molecular profiling of driver events in metastatic uveal melanoma. Nat Commun 11, 60. 10.1038/s41467-020-15606-0 PMC7171146 32313009

[11] Ewens KG , Kanetsky PA , Richards‐Yutz J , Purrazzella J , Shields CL , Ganguly T & Ganguly A (2014) Chromosome 3 status combined with BAP1 and EIF1AX mutation profiles are associated with metastasis in uveal melanoma. Invest Ophthalmol vis Sci 55, 5160–5167. 24970262 10.1167/iovs.14-14550

[12] Danset M , Milley S , Harou O , Vasseur D , Amini‐Adle M , Thomas L , Dalle S , Balme B & Lopez J (2020) Concomitant GNA11 and SF3B1 mutations in two cases of melanoma associated with blue naevus. Clin Exp Dermatol 45, 123–126. 31338849 10.1111/ced.14036

[13] Griewank KG , Muller H , Jackett LA , Emberger M , Moller I , van de Nes JA , Zimmer L , Livingstone E , Wiesner T , Scholz SL *et al*. (2017) SF3B1 and BAP1 mutations in blue nevus‐like melanoma. Mod Pathol 30, 928–939. 28409567 10.1038/modpathol.2017.23 PMC5533514

[14] Griewank KG , Koelsche C , van de Nes JAP , Schrimpf D , Gessi M , Moller I , Sucker A , Scolyer RA , Buckland ME , Murali R *et al*. (2018) Integrated genomic classification of melanocytic tumors of the central nervous system using mutation analysis, copy number alterations, and DNA methylation profiling. Clin Cancer Res 24, 4494–4504. 29891723 10.1158/1078-0432.CCR-18-0763

[15] Jensen DE , Proctor M , Marquis ST , Gardner HP , Ha SI , Chodosh LA , Ishov AM , Tommerup N , Vissing H , Sekido Y *et al*. (1998) BAP1: a novel ubiquitin hydrolase which binds to the BRCA1 RING finger and enhances BRCA1‐mediated cell growth suppression. Oncogene 16, 1097–1112. 9528852 10.1038/sj.onc.1201861

[16] Dey A , Seshasayee D , Noubade R , French DM , Liu J , Chaurushiya MS , Kirkpatrick DS , Pham VC , Lill JR , Bakalarski CE *et al*. (2012) Loss of the tumor suppressor BAP1 causes myeloid transformation. Science 337, 1541–1546. 22878500 10.1126/science.1221711 PMC5201002

[17] Ji Z , Mohammed H , Webber A , Ridsdale J , Han N , Carroll JS & Sharrocks AD (2014) The forkhead transcription factor FOXK2 acts as a chromatin targeting factor for the BAP1‐containing histone deubiquitinase complex. Nucleic Acids Res 42, 6232–6242. 24748658 10.1093/nar/gku274 PMC4041447

[18] Yu H , Mashtalir N , Daou S , Hammond‐Martel I , Ross J , Sui G , Hart GW , Rauscher FJ 3rd , Drobetsky E , Milot E *et al*. (2010) The ubiquitin carboxyl hydrolase BAP1 forms a ternary complex with YY1 and HCF‐1 and is a critical regulator of gene expression. Mol Cell Biol 30, 5071–5085. 20805357 10.1128/MCB.00396-10 PMC2953049

[19] Campagne A , Lee MK , Zielinski D , Michaud A , Le Corre S , Dingli F , Chen H , Shahidian LZ , Vassilev I , Servant N *et al*. (2019) BAP1 complex promotes transcription by opposing PRC1‐mediated H2A ubiquitylation. Nat Commun 10, 348. 30664650 10.1038/s41467-018-08255-x PMC6341105

[20] Weake VM & Workman JL (2008) Histone ubiquitination: triggering gene activity. Mol Cell 29, 653–663. 18374642 10.1016/j.molcel.2008.02.014

[21] Carbone M , Harbour JW , Brugarolas J , Bononi A , Pagano I , Dey A , Krausz T , Pass HI , Yang H & Gaudino G (2020) Biological mechanisms and clinical significance of BAP1 mutations in human cancer. Cancer Discov 10, 1103–1120. 32690542 10.1158/2159-8290.CD-19-1220 PMC8006752

[22] Matatall KA , Agapova OA , Onken MD , Worley LA , Bowcock AM & Harbour JW (2013) BAP1 deficiency causes loss of melanocytic cell identity in uveal melanoma. BMC Cancer 13, 371. 23915344 10.1186/1471-2407-13-371 PMC3846494

[23] Golas MM , Sander B , Will CL , Luhrmann R & Stark H (2003) Molecular architecture of the multiprotein splicing factor SF3b. Science 300, 980–984. 12738865 10.1126/science.1084155

[24] Furney SJ , Pedersen M , Gentien D , Dumont AG , Rapinat A , Desjardins L , Turajlic S , Piperno‐Neumann S , de la Grange P , Roman‐Roman S *et al*. (2013) SF3B1 mutations are associated with alternative splicing in uveal melanoma. Cancer Discov 3, 1122–1129. 23861464 10.1158/2159-8290.CD-13-0330 PMC5321577

[25] Jager MJ , Shields CL , Cebulla CM , Abdel‐Rahman MH , Grossniklaus HE , Stern MH , Carvajal RD , Belfort RN , Jia R , Shields JA *et al*. (2020) ) Uveal melanoma. Nat Rev Dis Primers 6, 24. 32273508 10.1038/s41572-020-0158-0

[26] Alsafadi S , Houy A , Battistella A , Popova T , Wassef M , Henry E , Tirode F , Constantinou A , Piperno‐Neumann S , Roman‐Roman S *et al*. (2016) Cancer‐associated SF3B1 mutations affect alternative splicing by promoting alternative branchpoint usage. Nat Commun 7, 10615. 26842708 10.1038/ncomms10615 PMC4743009

[27] Hakiri S , Osada H , Ishiguro F , Murakami H , Murakami‐Tonami Y , Yokoi K & Sekido Y (2015) Functional differences between wild‐type and mutant‐type BRCA1‐associated protein 1 tumor suppressor against malignant mesothelioma cells. Cancer Sci 106, 990–999. 26011428 10.1111/cas.12698 PMC4556387

[28] Deng Q , Li Y , Tedesco D , Liao R , Fuhrmann G & Sun P (2005) The ability of E1A to rescue ras‐induced premature senescence and confer transformation relies on inactivation of both p300/CBP and Rb family proteins. Cancer Res 65, 8298–8307. 16166306 10.1158/0008-5472.CAN-05-0054

[29] Ran FA , Hsu PD , Wright J , Agarwala V , Scott DA & Zhang F (2013) Genome engineering using the CRISPR‐Cas9 system. Nat Protoc 8, 2281–2308. 24157548 10.1038/nprot.2013.143 PMC3969860

[30] Zhou Q , Derti A , Ruddy D , Rakiec D , Kao I , Lira M , Gibaja V , Chan H , Yang Y , Min J *et al*. (2015) A chemical genetics approach for the functional assessment of novel cancer genes. Cancer Res 75, 1949–1958. 25788694 10.1158/0008-5472.CAN-14-2930

[31] Bray NL , Pimentel H , Melsted P & Pachter L (2016) Near‐optimal probabilistic RNA‐seq quantification. Nat Biotechnol 34, 525–527. 27043002 10.1038/nbt.3519

[32] Soneson C , Love MI & Robinson MD (2015) Differential analyses for RNA‐seq: transcript‐level estimates improve gene‐level inferences. F1000Res 4, 1521. 26925227 10.12688/f1000research.7563.1 PMC4712774

[33] Love MI , Huber W & Anders S (2014) Moderated estimation of fold change and dispersion for RNA‐seq data with DESeq2. Genome Biol 15, 550. 25516281 10.1186/s13059-014-0550-8 PMC4302049

[34] Kuilman T , Michaloglou C , Mooi WJ & Peeper DS (2010) The essence of senescence. Genes Dev 24, 2463–2479. 21078816 10.1101/gad.1971610 PMC2975923

[35] Chen L , Chen JY , Huang YJ , Gu Y , Qiu J , Qian H , Shao C , Zhang X , Hu J , Li H *et al*. (2018) The Augmented R‐Loop is a unifying mechanism for myelodysplastic syndromes induced by high‐risk splicing factor mutations. Mol Cell 69, 412–425. 29395063 10.1016/j.molcel.2017.12.029 PMC5957072

[36] van der Ent W , Burrello C , Teunisse AF , Ksander BR , van der Velden PA , Jager MJ , Jochemsen AG & Snaar‐Jagalska BE (2014) Modeling of human uveal melanoma in zebrafish xenograft embryos. Invest Ophthalmol vis Sci 55, 6612–6622. 25249605 10.1167/iovs.14-15202

[37] Yu H , Pak H , Hammond‐Martel I , Ghram M , Rodrigue A , Daou S , Barbour H , Corbeil L , Hebert J , Drobetsky E *et al*. (2014) Tumor suppressor and deubiquitinase BAP1 promotes DNA double‐strand break repair. Proc Natl Acad Sci USA 111, 285–290. 24347639 10.1073/pnas.1309085110 PMC3890818

[38] Te Raa GD , Derks IA , Navrkalova V , Skowronska A , Moerland PD , van Laar J , Oldreive C , Monsuur H , Trbusek M , Malcikova J *et al*. (2015) The impact of SF3B1 mutations in CLL on the DNA‐damage response. Leukemia 29, 1133–1142. 25371178 10.1038/leu.2014.318

[39] Klusmann I , Wohlberedt K , Magerhans A , Teloni F , Korbel JO , Altmeyer M & Dobbelstein M (2018) Chromatin modifiers Mdm2 and RNF2 prevent RNA:DNA hybrids that impair DNA replication. Proc Natl Acad Sci USA 115, E11311–E11320. 30413623 10.1073/pnas.1809592115 PMC6275510

[40] Khalili JS , Yu X , Wang J , Hayes BC , Davies MA , Lizee G , Esmaeli B & Woodman SE (2012) Combination small molecule MEK and PI3K inhibition enhances uveal melanoma cell death in a mutant GNAQ‐ and GNA11‐dependent manner. Clin Cancer Res 18, 4345–4355. 22733540 10.1158/1078-0432.CCR-11-3227 PMC3935730

[41] Paolella BR , Gibson WJ , Urbanski LM , Alberta JA , Zack TI , Bandopadhayay P , Nichols CA , Agarwalla PK , Brown MS , Lamothe R *et al*. (2017) Copy‐number and gene dependency analysis reveals partial copy loss of wild‐type SF3B1 as a novel cancer vulnerability. Elife 6, 68. 10.7554/eLife.23268 PMC5357138 28177281

[42] Tschentscher F , Husing J , Holter T , Kruse E , Dresen IG , Jockel KH , Anastassiou G , Schilling H , Bornfeld N , Horsthemke B *et al*. (2003) Tumor classification based on gene expression profiling shows that uveal melanomas with and without monosomy 3 represent two distinct entities. Cancer Res 63, 2578–2584. 12750282

[43] Harbour JW (2014) A prognostic test to predict the risk of metastasis in uveal melanoma based on a 15‐gene expression profile. Methods Mol Biol 1102, 427–440. 10.1007/978-1-62703-727-3_22. 24258991 PMC4476294

[44] O'Neil NJ , Bailey ML & Hieter P (2017) Synthetic lethality and cancer. Nat Rev Genet 18, 613–623. 28649135 10.1038/nrg.2017.47

[45] Behan FM , Iorio F , Picco G , Goncalves E , Beaver CM , Migliardi G , Santos R , Rao Y , Sassi F , Pinnelli M *et al*. (2019) Prioritization of cancer therapeutic targets using CRISPR‐Cas9 screens. Nature 568, 511–516. 30971826 10.1038/s41586-019-1103-9

[46] Chan EM , Shibue T , McFarland JM , Gaeta B , Ghandi M , Dumont N , Gonzalez A , McPartlan JS , Li T , Zhang Y *et al*. (2019) WRN helicase is a synthetic lethal target in microsatellite unstable cancers. Nature 568, 551–556. 30971823 10.1038/s41586-019-1102-x PMC6580861

[47] McDonald ER 3rd , de Weck A , Schlabach MR , Billy E , Mavrakis KJ , Hoffman GR , Belur D , Castelletti D , Frias E , Gampa K *et al*. (2017) Project DRIVE: a compendium of cancer dependencies and synthetic lethal relationships uncovered by large‐scale. Deep RNAi Screening. Cell 170, e510. 10.1016/j.cell.2017.07.005 28753431

[48] Tsherniak A , Vazquez F , Montgomery PG , Weir BA , Kryukov G , Cowley GS , Gill S , Harrington WF , Pantel S , Krill‐Burger JM *et al*. (2017) Defining a cancer dependency map. Cell 170, 564–576. 28753430 10.1016/j.cell.2017.06.010 PMC5667678

[49] Huang A , Garraway LA , Ashworth A & Weber B (2020) Synthetic lethality as an engine for cancer drug target discovery. Nat Rev Drug Discov 19, 23–38. 31712683 10.1038/s41573-019-0046-z

[50] Li S , Topatana W , Juengpanich S , Cao J , Hu J , Zhang B , Ma D , Cai X & Chen M (2020) Development of synthetic lethality in cancer: molecular and cellular classification. Signal Transduct Target Ther 5, 241. 33077733 10.1038/s41392-020-00358-6 PMC7573576

[51] Lee SC , North K , Kim E , Jang E , Obeng E , Lu SX , Liu B , Inoue D , Yoshimi A , Ki M *et al*. (2018) Synthetic lethal and convergent biological effects of cancer‐associated spliceosomal gene mutations. Cancer Cell 34, 225–241. 30107174 10.1016/j.ccell.2018.07.003 PMC6373472

[52] Morel D , Almouzni G , Soria JC & Postel‐Vinay S (2017) Targeting chromatin defects in selected solid tumors based on oncogene addiction, synthetic lethality and epigenetic antagonism. Ann Oncol 28, 254–269. 28426098 10.1093/annonc/mdw552

[53] Oike T , Ogiwara H , Tominaga Y , Ito K , Ando O , Tsuta K , Mizukami T , Shimada Y , Isomura H , Komachi M *et al*. (2013) A synthetic lethality‐based strategy to treat cancers harboring a genetic deficiency in the chromatin remodeling factor BRG1. Cancer Res 73, 5508–5518. 23872584 10.1158/0008-5472.CAN-12-4593

[54] Tang YC , Ho SC , Tan E , Ng AWT , McPherson JR , Goh GYL , Teh BT , Bard F & Rozen SG (2018) Functional genomics identifies specific vulnerabilities in PTEN‐deficient breast cancer. Breast Cancer Res 20, 22. 29566768 10.1186/s13058-018-0949-3 PMC5863852

[55] Unni AM , Lockwood WW , Zejnullahu K , Lee‐Lin SQ & Varmus H (2015) Evidence that synthetic lethality underlies the mutual exclusivity of oncogenic KRAS and EGFR mutations in lung adenocarcinoma. Elife 4, e06907. 26047463 10.7554/eLife.06907 PMC4478584

[56] Zhao D , Lu X , Wang G , Lan Z , Liao W , Li J , Liang X , Chen JR , Shah S , Shang X *et al*. (2017) Synthetic essentiality of chromatin remodelling factor CHD1 in PTEN‐deficient cancer. Nature 542, 484–488. 28166537 10.1038/nature21357 PMC5448706

[57] Li H , Liu ZY , Wu N , Chen YC , Cheng Q & Wang J (2020) PARP inhibitor resistance: the underlying mechanisms and clinical implications. Mol Cancer 19, 107. 32563252 10.1186/s12943-020-01227-0 PMC7305609

[58] Gao J , Aksoy BA , Dogrusoz U , Dresdner G , Gross B , Sumer SO , Sun Y , Jacobsen A , Sinha R , Larsson E *et al*. (2013) Integrative analysis of complex cancer genomics and clinical profiles using the cBioPortal. Sci Signal 6, p11. 10.1126/scisignal.2004088 PMC4160307 23550210

