# Supplementary figures and images for: Co‐occurrence of BAP1 and SF3B1 mutations in uveal melanoma induces cellular senescence
Source: Mol Oncol. 2021 Nov 12;16(3):607–29. doi: 10.1002/1878-0261.13128 (PMC8807356; doi:10.1002/1878-0261.13128)

Fig.S1

A

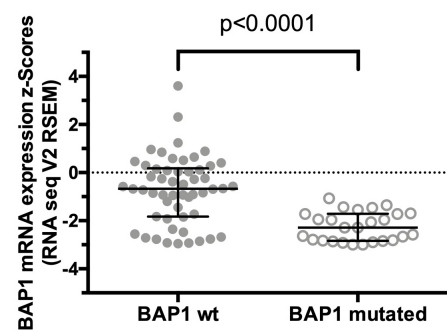

B

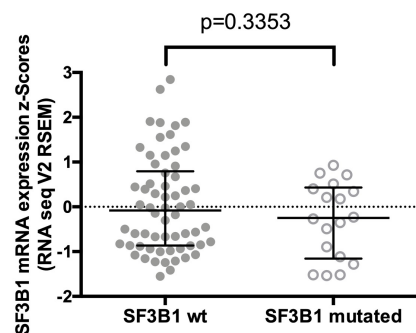

C

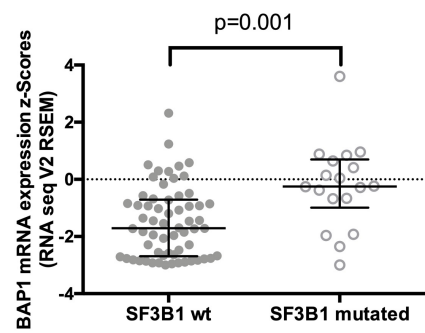

Supplement: Supplementary file 1 — Fig. S1. mRNA expression of BRCA1‐associated protein 1 (BAP1) and splicing factor 3B subunit 1 (SF3B1) in TCGA uveal melanoma (UM) dataset. [file MOL2-16-607-s022.pdf]

Fig.S2

A

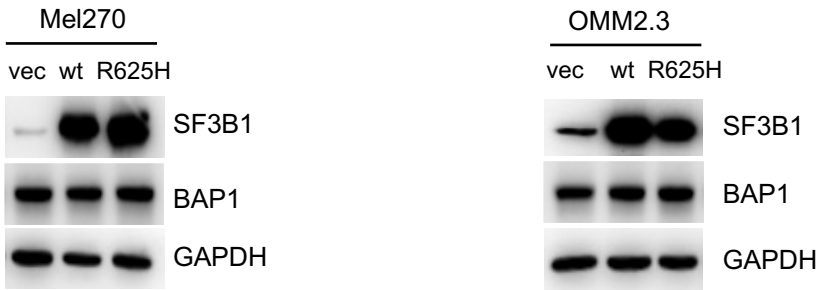

B

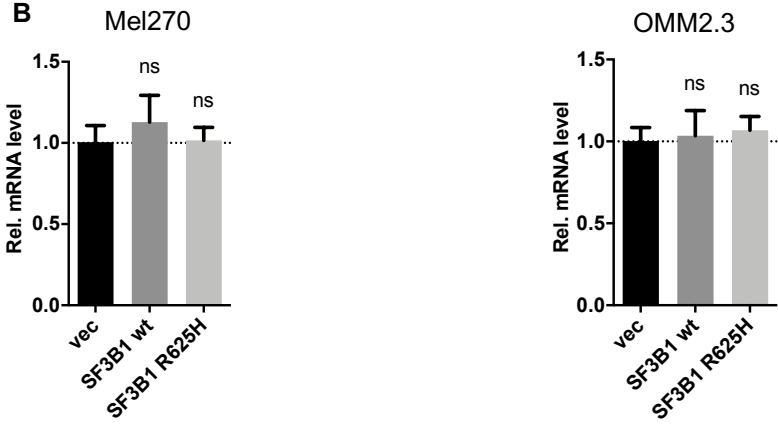

Supplement: Supplementary file 2 — Fig S2. Ectopic expression of either wild‐type or mutant SF3B1 did not alter BAP1 expression in Mel270 and OMM2.3 cells. [file MOL2-16-607-s019.pdf]

Fig.S4

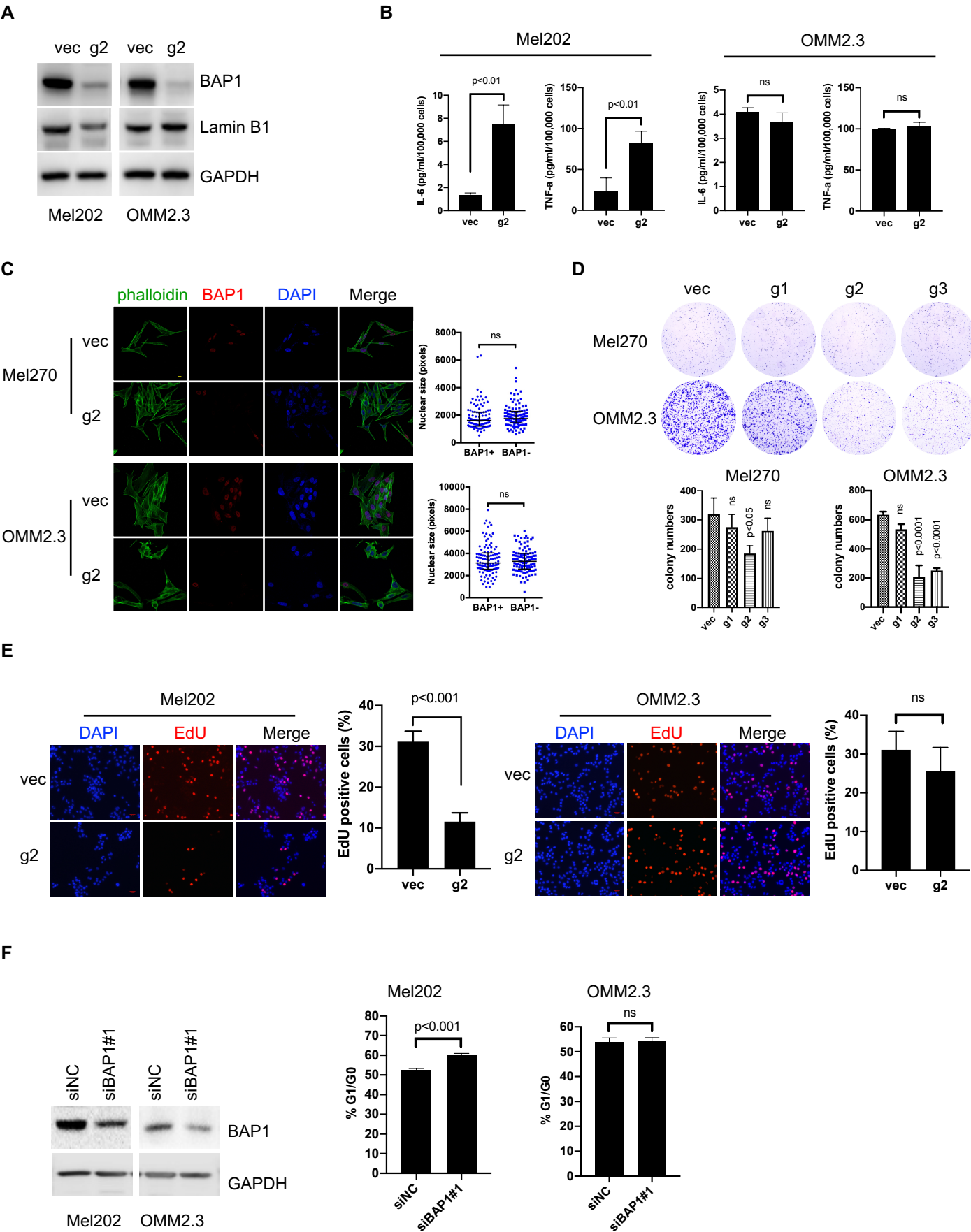

Supplement: Supplementary file 4 — Fig S4. BAP1 deficiency in UM cells with mutant SF3B1 induces senescent phenotype. [file MOL2-16-607-s016.pdf]

Fig.S7

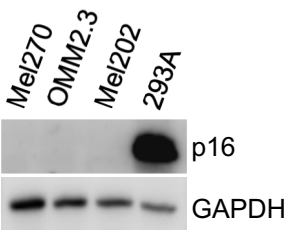

Supplement: Supplementary file 7 — Fig S7. The expression of p16 INK4A is absent in Mel270, OMM2.3 and Mel202 cells. [file MOL2-16-607-s011.pdf]

Fig.S9

A

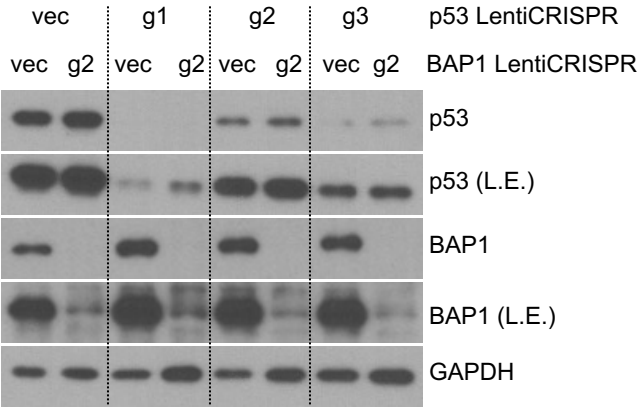

B

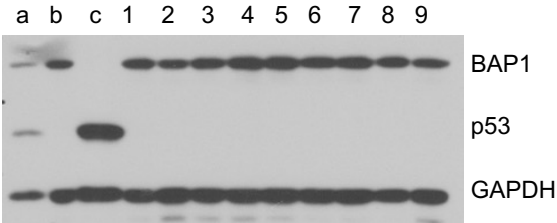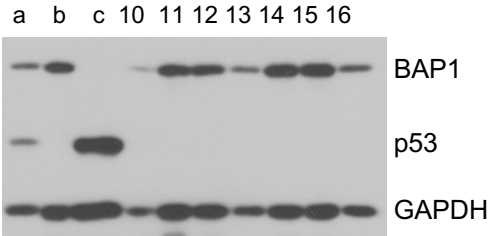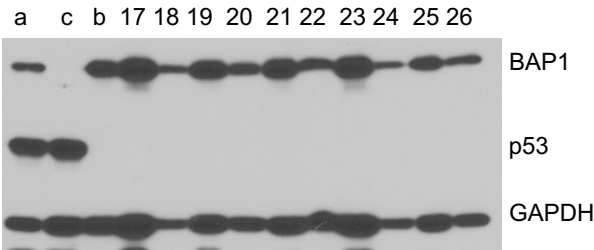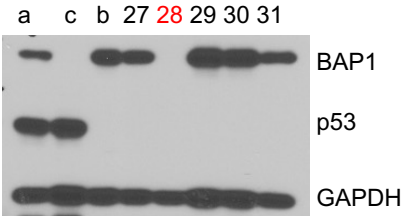

C

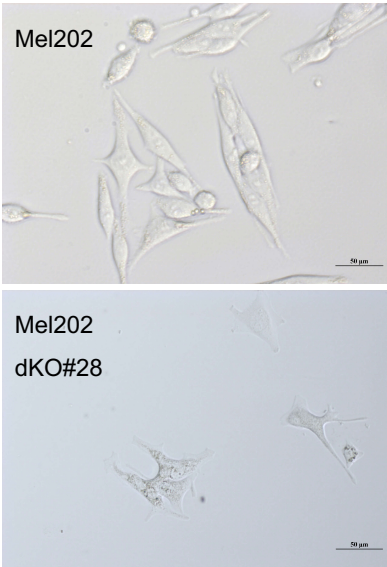

Supplement: Supplementary file 9 — Fig S9. Identification and characterization of BAP1 and TP53 double knockout clone from Mel202 cells. [file MOL2-16-607-s014.pdf]

Fig.S10

A

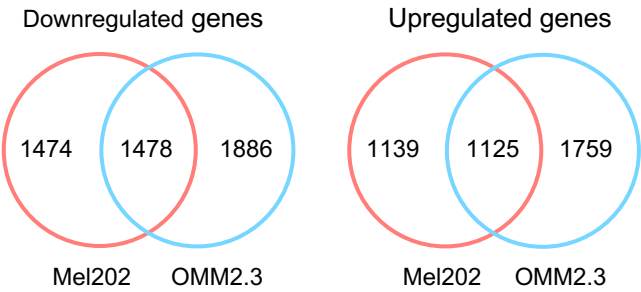

B

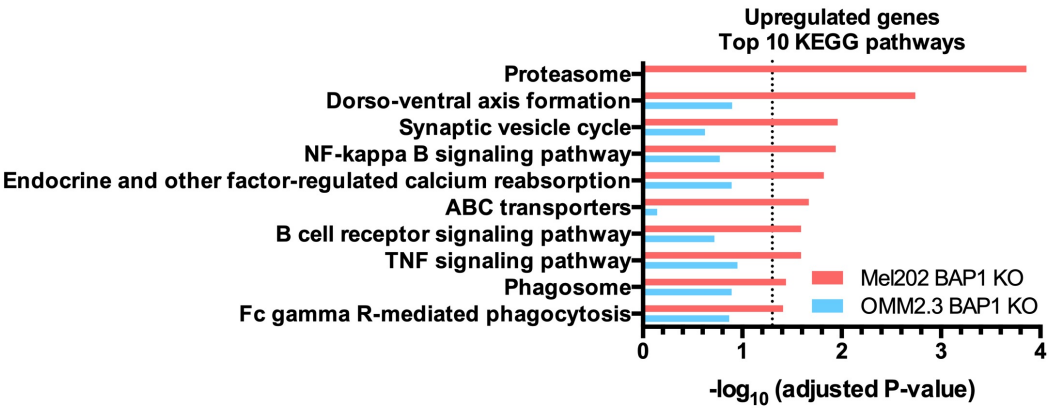

Supplement: Supplementary file 10 — Fig S10. KEGG pathways enrichment analysis for the identified DEGs. [file MOL2-16-607-s004.pdf]

Fig.S11

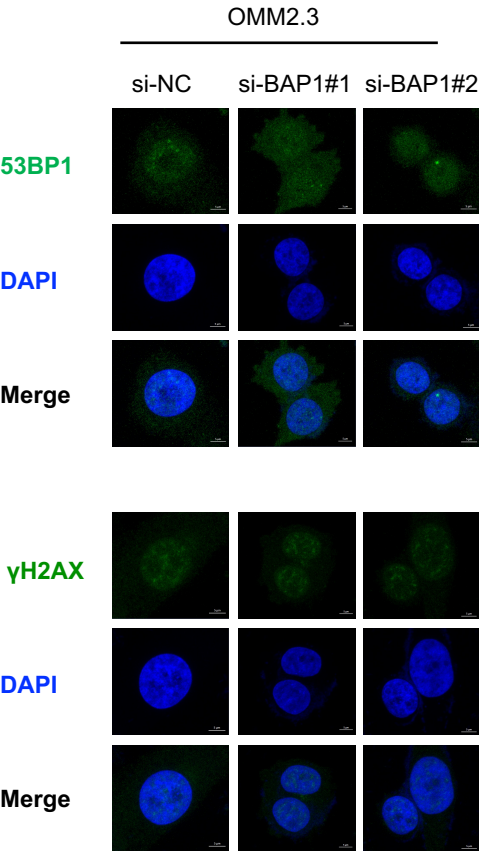

Supplement: Supplementary file 11 — Fig S11. BAP1 knockdown does not cause DNA damage in OMM2.3 cells. [file MOL2-16-607-s021.pdf]

Fig.S13

A

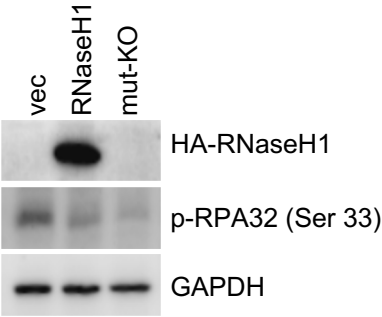

B

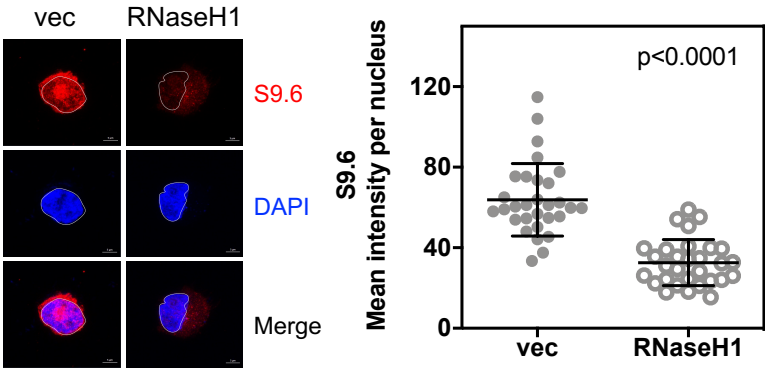

C

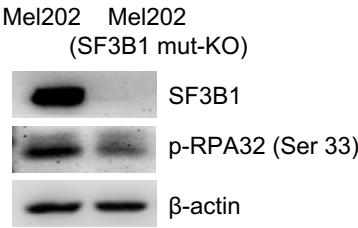

D

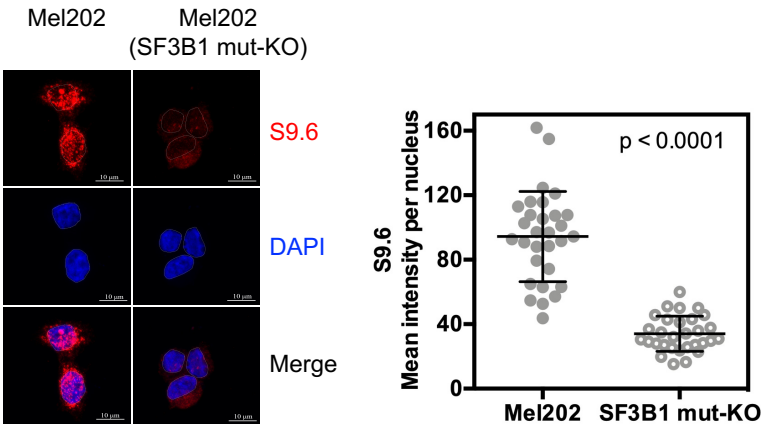

Supplement: Supplementary file 13 — Fig S13. Deletion of mutant SF3B1 in Mel202 cells alleviates R‐loop formation. [file MOL2-16-607-s007.pdf]

Fig.S14

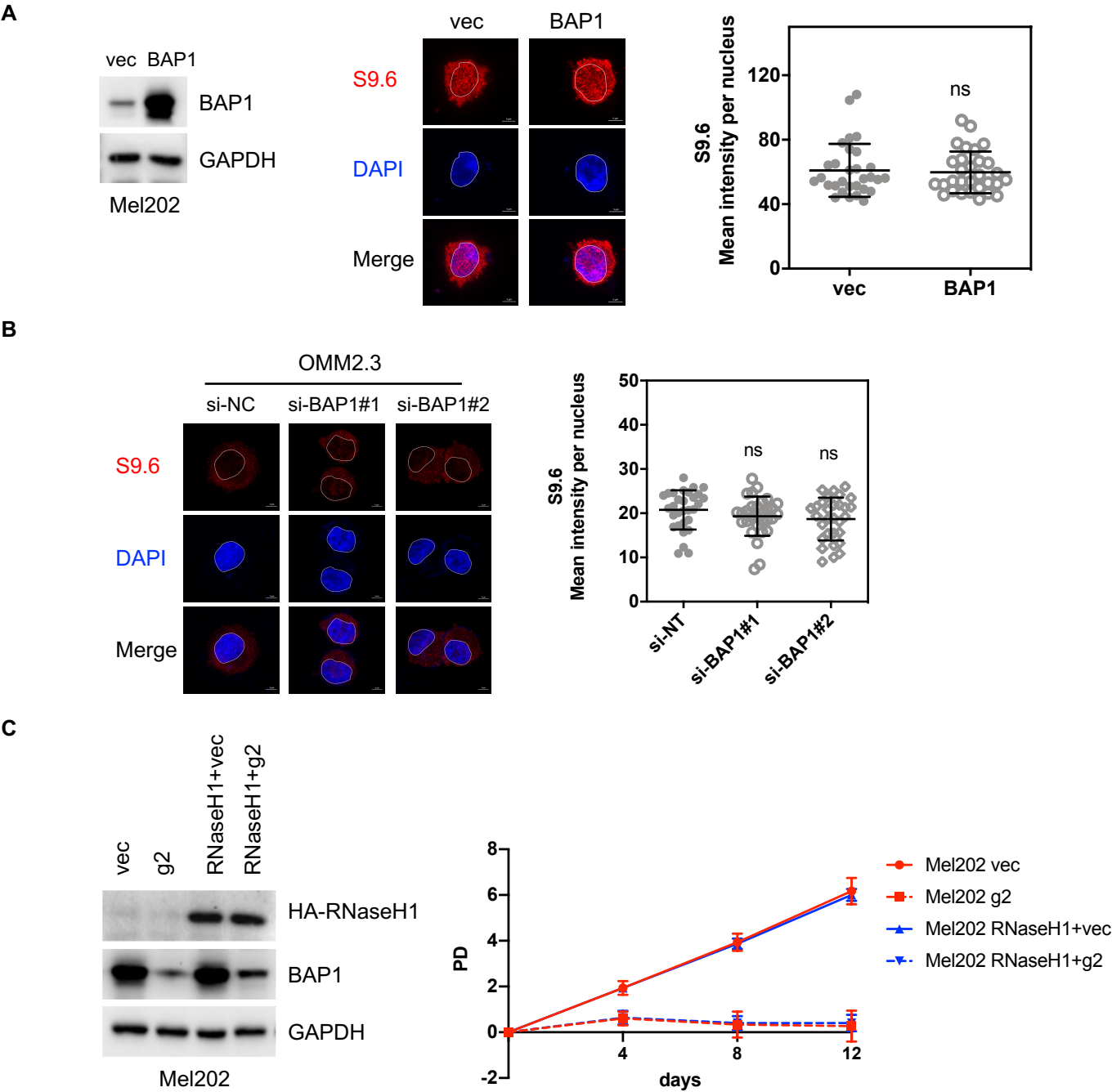

Supplement: Supplementary file 14 — Fig S14. Alleviation of R‐loops does not rescue BAP1 deletion‐induced growth arrest in Mel202 cells. [file MOL2-16-607-s018.pdf]

Fig.S15

A

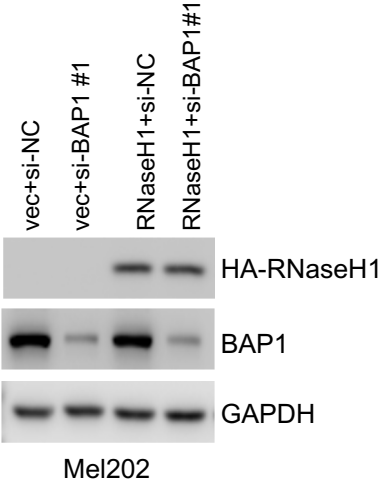

B

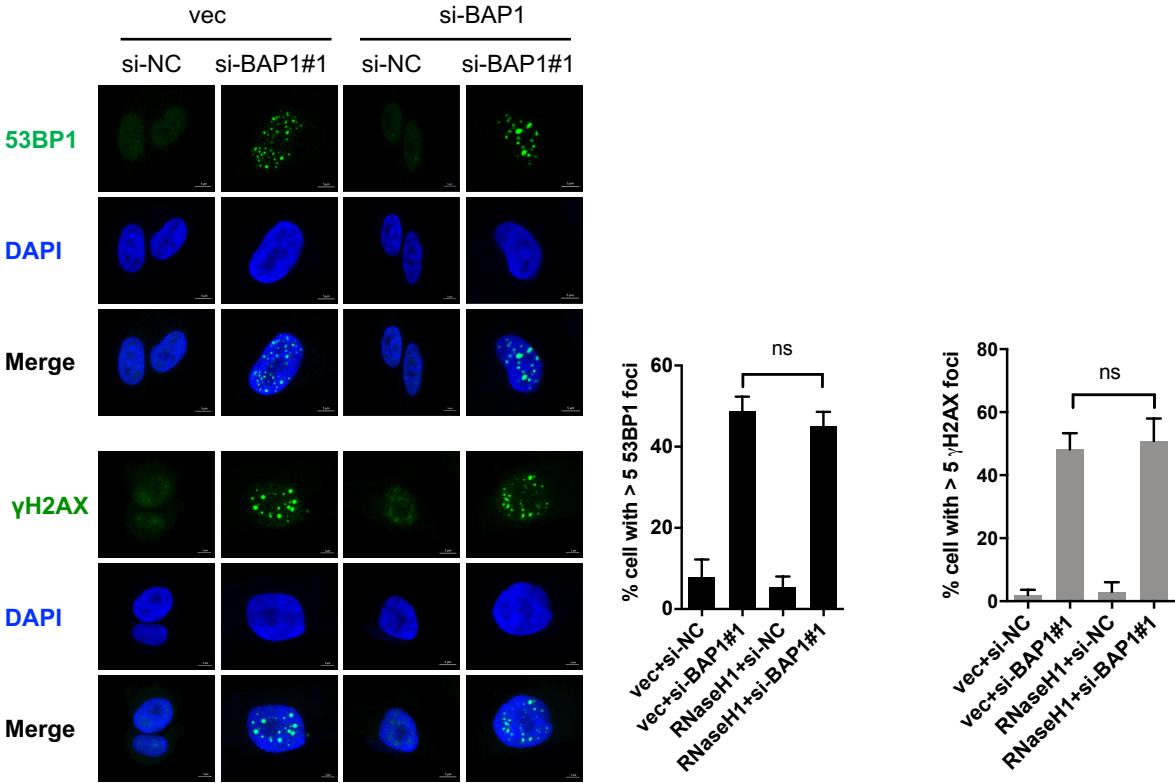

Supplement: Supplementary file 15 — Fig S15. Alleviation of R‐loops does not attenuate BAP1 loss‐induced DNA damage in Mel202 cells. [file MOL2-16-607-s001.pdf]

Fig.S16

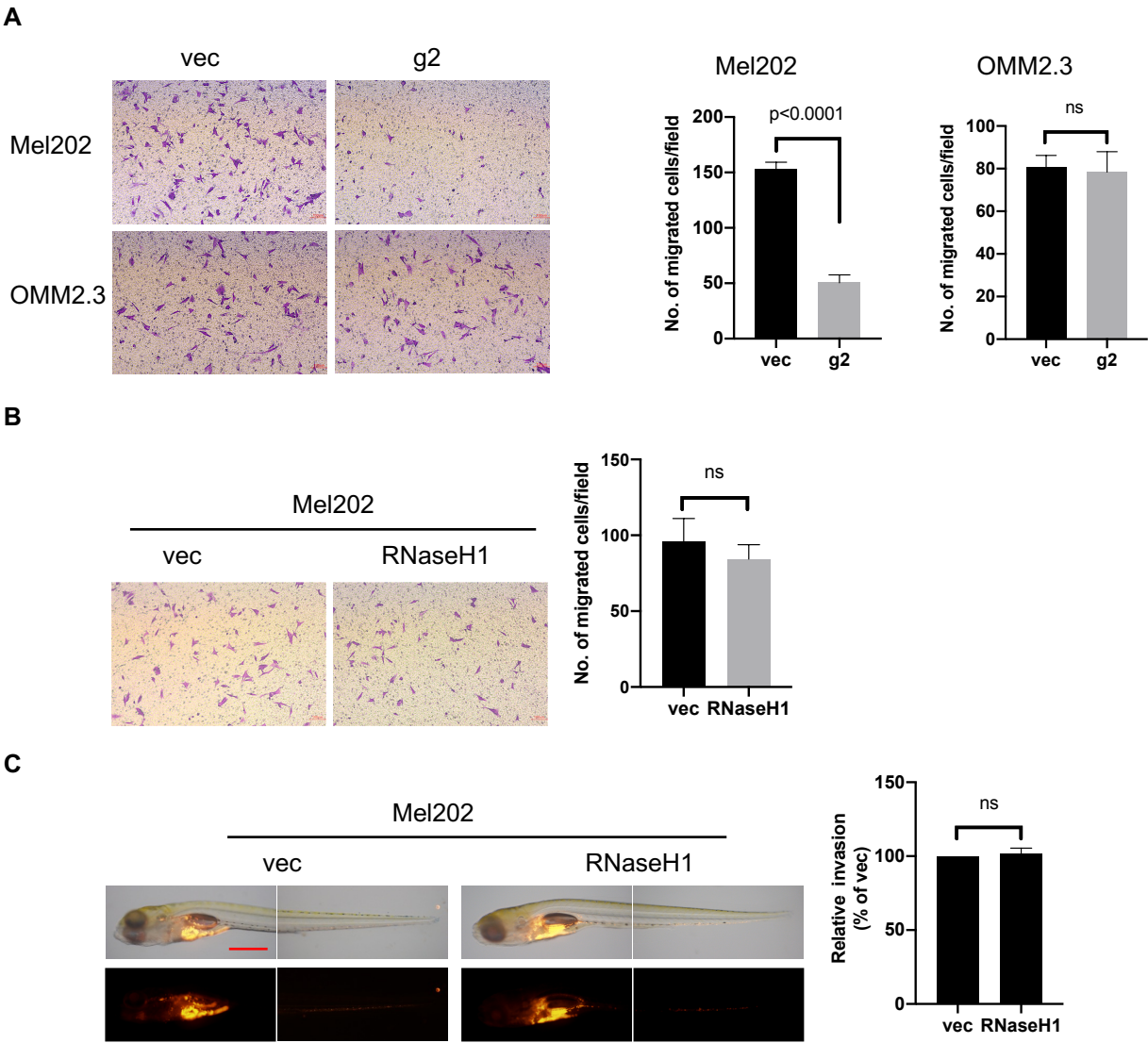

Supplement: Supplementary file 16 — Fig S16. BAP1 deletion decreases cell migration in Mel202 but not OMM2.3 cells. [file MOL2-16-607-s003.pdf]

Fig.S17

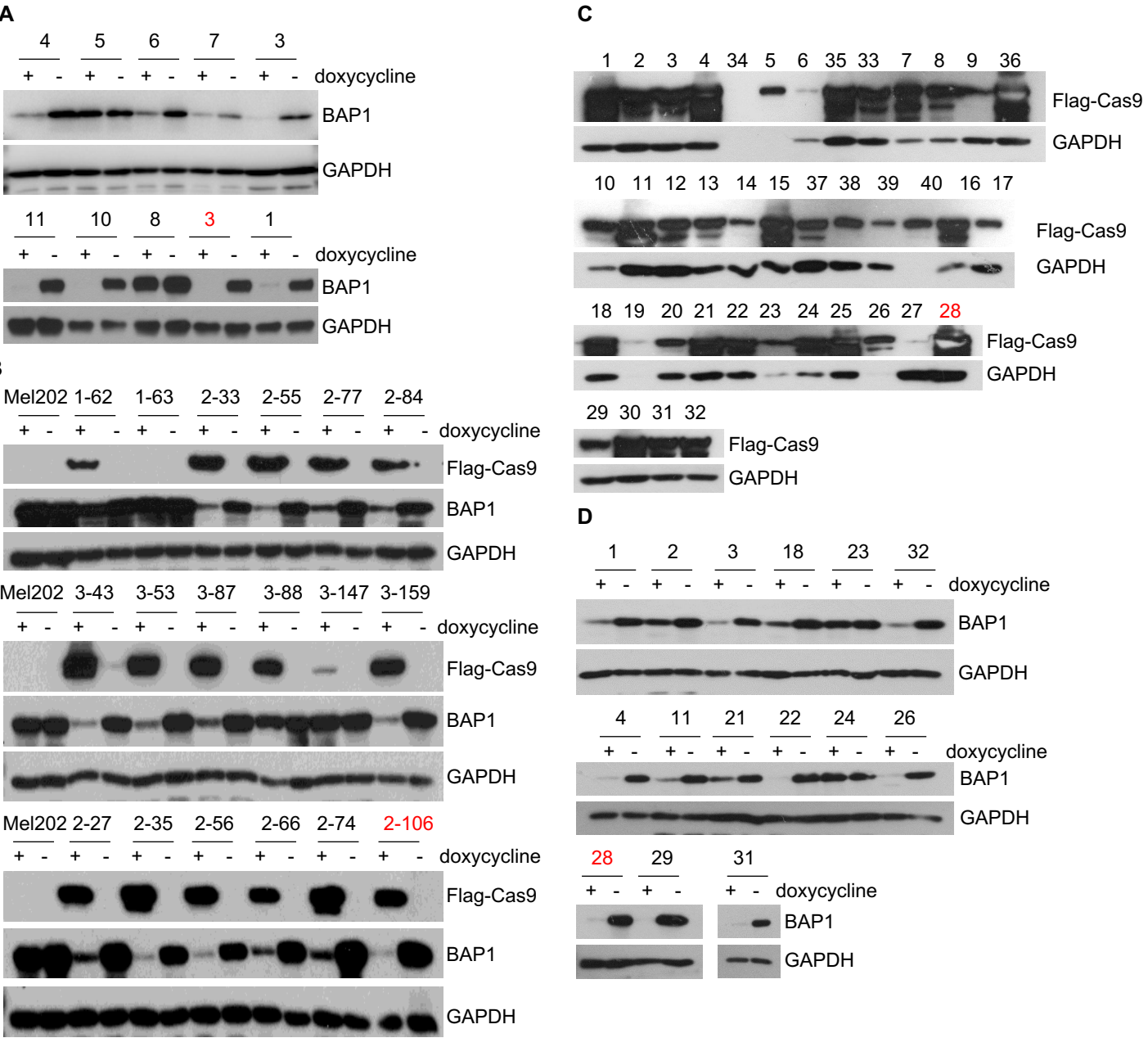

Supplement: Supplementary file 17 — Fig S17. Screening doxycycline‐inducible BAP1 KO clones. [file MOL2-16-607-s015.pdf]

Fig.S18

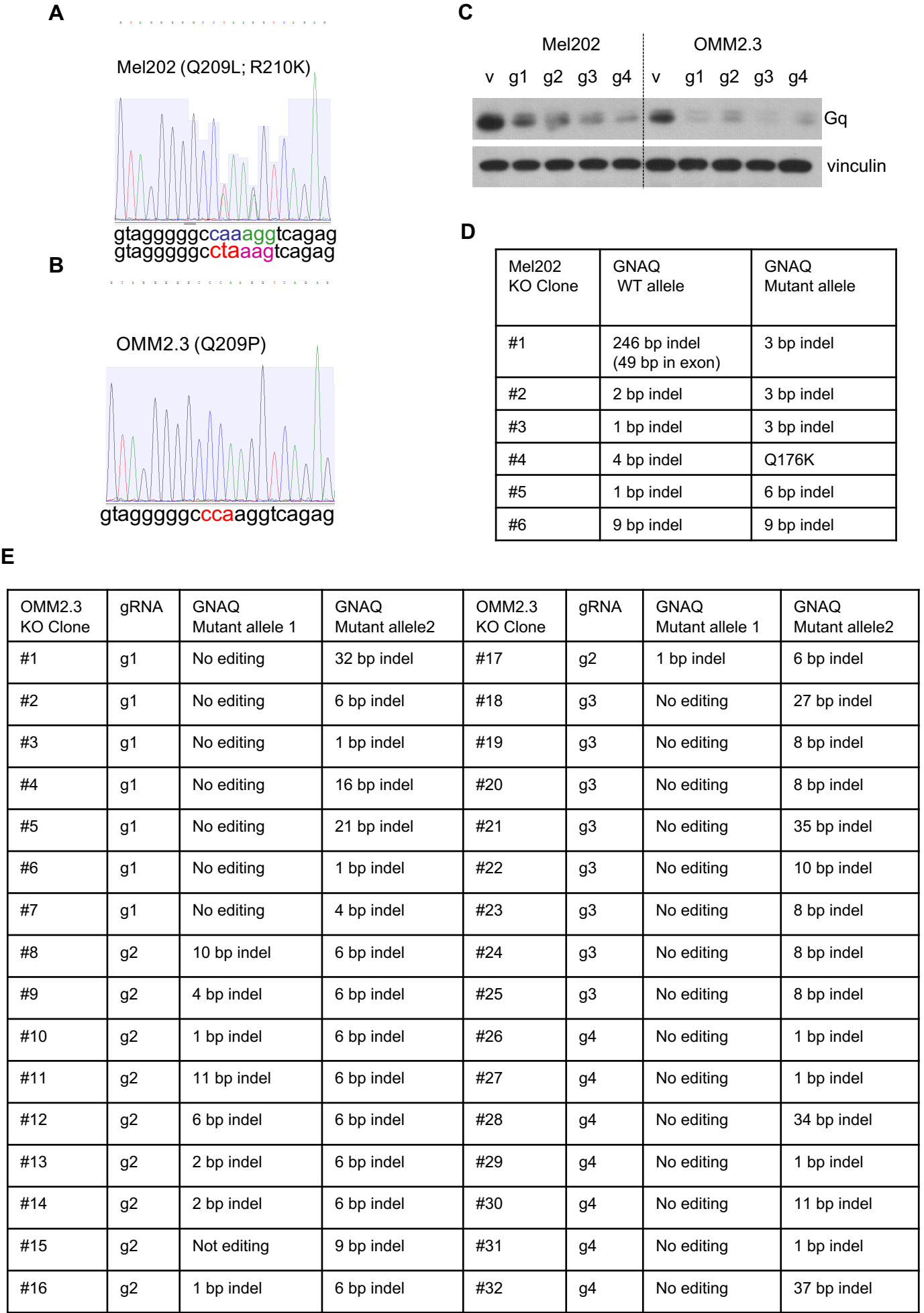

Supplement: Supplementary file 18 — Fig S18. Characterization of genomic editing of GNAQ by CRISPR‐Cas9. [file MOL2-16-607-s009.pdf]
