# Supplementary material for: Co‐occurrence of BAP1 and SF3B1 mutations in uveal melanoma induces cellular senescence
Source: Mol Oncol. 2021 Nov 12;16(3):607–29. doi: 10.1002/1878-0261.13128 (PMC8807356; doi:10.1002/1878-0261.13128)

Fig.S3

A

Cell line	92.1	Mel202	Mel270	OMM2.2	OMM2.3	OMM2.5	OMM1	OCM1	Mel285	Mel290
GNAQ	Q209L	Q209L	Q209P	Q209P	Q209P	Q209P	WT	WT	WT	WT
GNA11	WT	WT	WT	WT	WT	WT	Q209L	WT	WT	WT
BRAF	WT	WT	WT	WT	WT	WT	WT	V600E	WT	WT
BAP1	WT	WT	WT	WT	WT	WT	WT	WT	WT	WT
SF3B1	WT	R625G	WT	WT	WT	WT	WT	WT	WT	WT
EIF1AX	G6D	WT	WT	WT	WT	WT	WT	WT	WT	WT

B

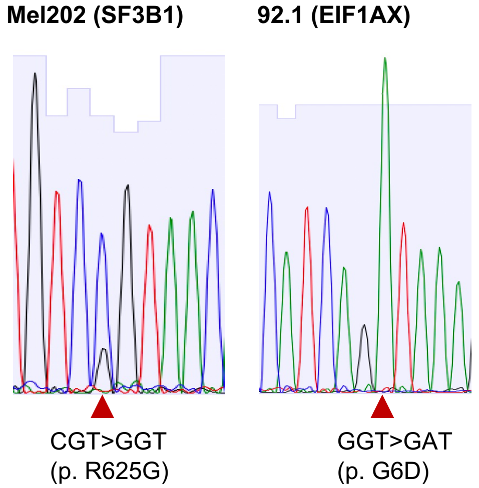

C

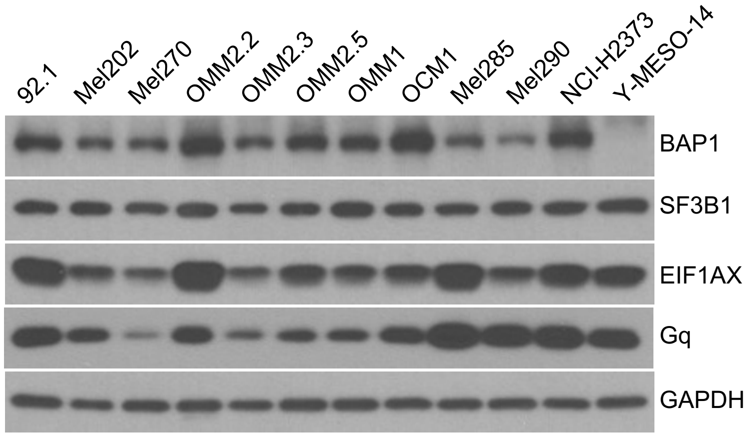

Supplement: Supplementary file 3 — Fig S3. Genomic mutations and protein expression of GNAQ, GNA11, BRAF, BAP1, SF3B1 and EIF1AX in multiple UM cell lines. [file MOL2-16-607-s002.pdf]
