# Supplementary material for: Co‐occurrence of BAP1 and SF3B1 mutations in uveal melanoma induces cellular senescence
Source: Mol Oncol. 2021 Nov 12;16(3):607–29. doi: 10.1002/1878-0261.13128 (PMC8807356; doi:10.1002/1878-0261.13128)
Supplement: Supplementary file 5 — Fig S5. Identification of BAP1 KO clones from Mel270 and OMM2.3 cells. [file MOL2-16-607-s005.pdf]

Fig.S5

A

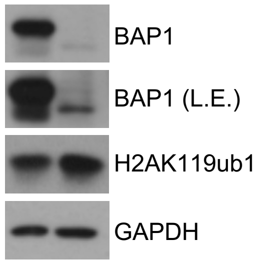

B

Guide RNA (BAP1 g2) 5'-ACCCACCCTGAGTCGCATGA-3'
Genomic DNA CGTGGACCTGGGACCCACCCTGAGTCGCATGAAGG
PAM

Mel270 BAP1 KO

KO#1 Allele 1 CGTGGACCTGGGACCCACCCTGAGTCAGCATGAAGG
Indel
KO#1 Allele 2 CGTGGACCTGGGACCCACCCTGAGT-2 bp-CATGAAGG
Indel

OMM2.3 BAP1 KO

KO#1 Allele 1 CGTGGA-49 bp-CCTGGGACCCACCCTGAGTCGCATGAAGG
Indel
KO#1 Allele 2 CGTGGACCTGGGACCCACCCTGTAGTCGCATGAAGG
Indel
KO#2 Allele 1 CGTGGACCTGGGACCCACCCTGAGCTTCGCATGAAGG
Indel
KO#2 Allele 2 CGTGGACCTGGGACCCACCCTGAGATCGCATGAAGG
Indel
