# Supplementary material for: Co‐occurrence of BAP1 and SF3B1 mutations in uveal melanoma induces cellular senescence
Source: Mol Oncol. 2021 Nov 12;16(3):607–29. doi: 10.1002/1878-0261.13128 (PMC8807356; doi:10.1002/1878-0261.13128)

Fig.S6

A

Guide RNA (SF3B1 g1) 5’-AAGATCGCCAAGACTCACGA-3’
Genomic DNA ATGGCGAAGATCGCCAAGACTCACGAAGGTAA
PAM

Mel202 SF3B1 mut-KO
Mutant allele ATGGCGAAGATCGCCAAGACTC-1 bp-CGAAGGTAA
Indel
Wild-type allele ATGGCGAAGATCGCCAAGACTCA-3 bp-AGGTAA
Indel
p.His8_Glu9delinsGln

C

Mel202 KO Clone	SF3B1 WT allele	SF3B1 Mutant allele
SF3B1 mut-KO	3 bp indel	1 bp indel
#1	6 bp indel	1 bp indel
#2	Missense mutation (H8P)	1 bp indel
#3	6 bp indel	1 bp indel
#4	3 bp indel	1 bp indel
#5	6 bp indel	6 bp indel

B

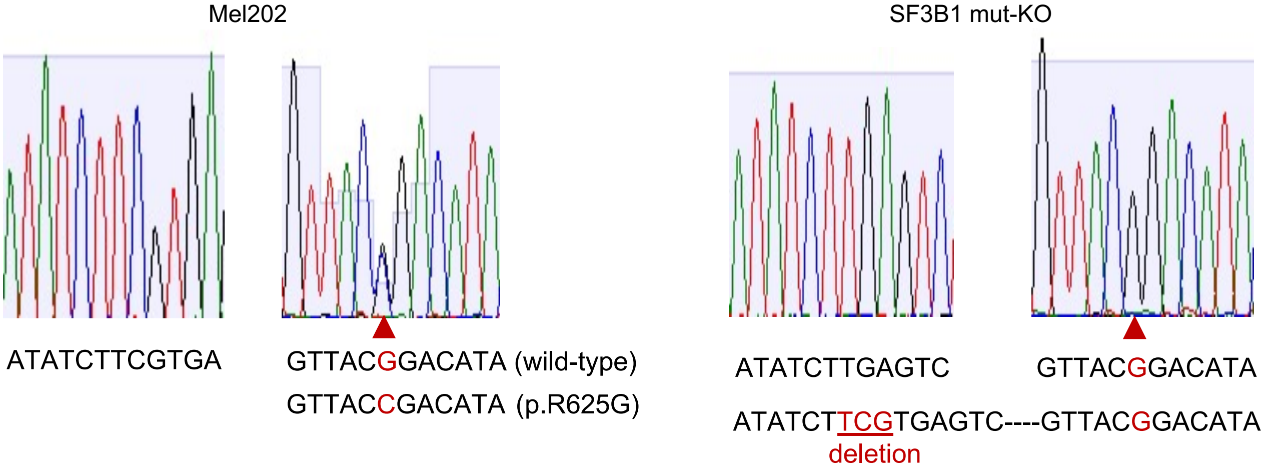

D

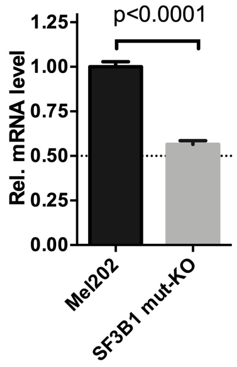

E

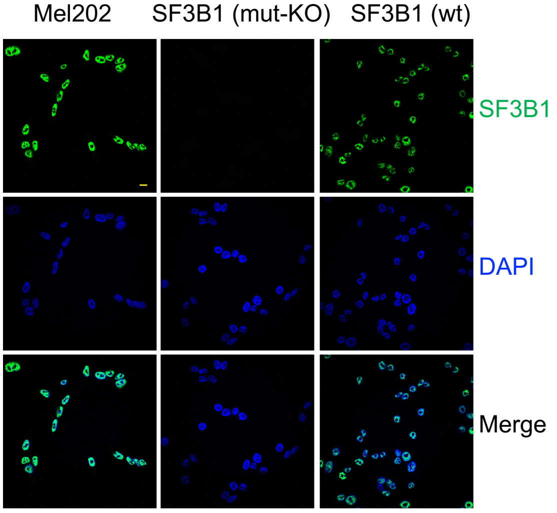

Supplement: Supplementary file 6 — Fig S6. Identification of Mel202 SF3B1 mut‐KO clone. [file MOL2-16-607-s012.pdf]
