# Supplementary material for: Co‐occurrence of BAP1 and SF3B1 mutations in uveal melanoma induces cellular senescence
Source: Mol Oncol. 2021 Nov 12;16(3):607–29. doi: 10.1002/1878-0261.13128 (PMC8807356; doi:10.1002/1878-0261.13128)
Supplement: Supplementary file 8 — Fig S8. Sanger DNA sequencing shows genomic editing of TP53 introduced by CRISPR‐Cas9 in Mel202 p53 KO clones (#1 and #2). [file MOL2-16-607-s010.pdf]

Fig.S8

Guide RNA (TP53 g1) 5’-CCATTGTTCAATATCGTCCG-3’
Genomic DNA GGGTCTTCAGTGAACCATTGTTCAATATCGTCCGGGGACA
PAM

Mel202 p53 KO

KO#1 Allele 1 GGGTCTTCAGTGAACCATTGTTCAATATCGTTCCGGGGACA
Indel
KO#1 Allele 2 GGGTCTTCAGTGAACCATTGTTCAATATCGTTCCGGGGACA
Indel
KO#2 Allele 1 GGGTCTTCAGTGAACCATTGTTCAATATCGTTCCGGGGACA
Indel
KO#2 Allele 2 GGGTCTTCAGTGAACCATTGTTCAATATCGTCCGGGGACA
GCATCAAATCATCCATTGCTTGGGACGGCAAGGGGGACTGTAGATGGGTG
78 bp deletion
