# Supplementary material for: Co‐occurrence of BAP1 and SF3B1 mutations in uveal melanoma induces cellular senescence
Source: Mol Oncol. 2021 Nov 12;16(3):607–29. doi: 10.1002/1878-0261.13128 (PMC8807356; doi:10.1002/1878-0261.13128)
Supplement: Supplementary file 12 — Fig S12. Effects of BAP1 KO on cell sensitivity to DNA damaging agents in OCM1 cells. [file MOL2-16-607-s020.pdf]

Fig.S12

A

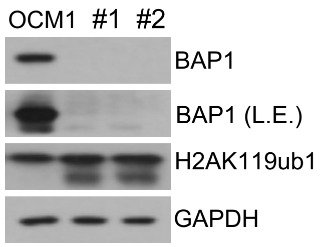

C

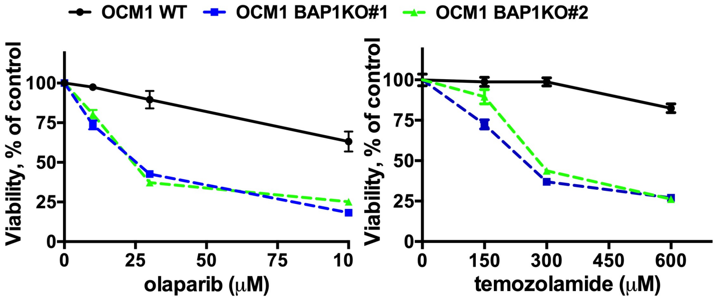

B

Guide RNA (BAP1 g2) 5'-ACCCACCCTGAGTCGCATGA-3'
Genomic DNA CGTGGACCTGGGACCCACCCTGAGTCGCATGA<u>AGG</u>ACTTCACCAAGGGTT
PAM

<u>OCM1</u>
KO#1 Allele 1 CGTGGACCTGGGACC------------------------26 bp----------------------CCAAGGGTT
Indel
KO#1 Allele 2 CGTGGACCTGGGACCCACCCTGAGTCG<u>A</u>CATGA<u>AGG</u>ACTTCACCAAGGGTT
Indel
KO#2 Allele 1 CGTGGACCTGGGACCCACCCTGAGTCGC<u>A</u>ATGA<u>AGG</u>ACTTCACCAAGGGTT
Indel
KO#2 Allele 2 CGTGGACCTGGGACCCACCCTGAGTCGC<u>A</u>ATGA<u>AGG</u>ACTTCACCAAGGGTT
Indel
