# Supplementary material for: Co‐occurrence of BAP1 and SF3B1 mutations in uveal melanoma induces cellular senescence
Source: Mol Oncol. 2021 Nov 12;16(3):607–29. doi: 10.1002/1878-0261.13128 (PMC8807356; doi:10.1002/1878-0261.13128)
Supplement: Supplementary file 19 — Table S1. Guide sequences used. [file MOL2-16-607-s023.doc]

Supplementary Table 1. Guide sequences used

| **sgRNA** | **CRISPR targeting Sequence** |
| --- | --- |
| BAP1 g1 | CGACCTTCAGAGCAAATGTC |
| BAP1 g2 | ACCCACCCTGAGTCGCATGA |
| BAP1 g3 | TGAAGTCCTTCATGCGACTC |
| SF3B1 g1 | AAGATCGCCAAGACTCACGA |
| SF3B1 g2 | CCGCTTACCTTCGTGAGTCT |
| SF3B1 g3 | GGAGTGGGCCTCGATTCTAC |
| TP53 g1 | CCATTGTTCAATATCGTCCG |
| TP53 g2 | GGGCAGCTACGGTTTCCGTC |
| TP53 g3 | TCCATTGCTTGGGACGGCAA |
| GNAQ g1 | CTAAGCACATCTTGTTGCGT |
| GNAQ g2 | GAACAATTATCACATACCCC |
| GNAQ g3 | ATCTTGTTGCGTAGGCAGGT |
| GNAQ g4 | CTTGCAGAATGGTCGATGTA |
| Non-targeting control gRNA | Addgene Cat #80173 |
