# Supplementary material for: Co‐occurrence of BAP1 and SF3B1 mutations in uveal melanoma induces cellular senescence
Source: Mol Oncol. 2021 Nov 12;16(3):607–29. doi: 10.1002/1878-0261.13128 (PMC8807356; doi:10.1002/1878-0261.13128)
Supplement: Supplementary file 20 — Table S2. Antibodies used. [file MOL2-16-607-s017.doc]

Supplementary Table 2. Antibodies used

| **Antibody** | **Company** | **Catalog** | **Dilution factor** |
| --- | --- | --- | --- |
| Mouse monoclonal anti-BAP1 | Santa Cruz Biotechnology | Cat# sc-28383,  RRID:AB_626723 | IF (1:100)  WB (1:200) |
| Rabbit monoclonal anti-SF3B1 | Cell Signaling Technology | Cat# 14434, RRID:AB_2798479 | IF (1:1000)  WB (1:1000) |
| Rabbit monoclonal anti-EIF1AX | Abcam | Cat# ab177939 | WB (1:10000) |
| Rabbit polyclonal anti-Gq | Santa Cruz Biotechnology | Cat# sc-393, RRID:AB_631536 | WB (1:200) |
| Mouse monoclonal anti-p53 | Santa Cruz Biotechnology | Cat# sc-126, RRID:AB_628082 | WB (1:200) |
| Rabbit polyclonal anti-phospho-p53 (Ser15) | Cell Signaling Technology | Cat #9284 | WB (1:1000) |
| Rabbit monoclonal anti-p21 | Cell Signaling Technology | Cat #2947 | WB (1:1000) |
| Rabbit monoclonal anti-PARP | Cell Signaling Technology | Cat #9532 | WB (1:1000) |
| Rabbit monoclonal anti-p-ATM (Ser1981) | Cell Signaling Technology | Cat #5883, RRID:AB_10835213 | WB (1:1000) |
| Rabbit monoclonal anti-ATM | Cell Signaling Technology | Cat #2873,  RRID:AB_2062659 | WB (1:1000) |
| Rabbit monoclonal anti-H2AK119ub1 | Cell Signaling Technology | Cat #8240 | WB (1:200000) |
| Mouse monoclonal anti-DNA-RNA hybrid | Merck Millipore | Cat #MABE1095 | IF (1:50) |
| Rabbit monoclonal anti-phospho-RPA32 (Ser33) | Bethyl | Cat #A300-246 | WB (1:1000) |
| Mouse monoclonal anti-vinculin | Sigma-Aldrich | Cat #V9131  RRID:AB_477629 | WB (1:10000) |
| Mouse monoclonal anti-Lamin B1 | Santa Cruz Biotechnology | Cat#sc-374015  RRID:AB_10947408 | WB (1:200) |
| Rabbit polyclonal anti-gamma H2A.X (phosphor Ser139) | Abcam | Cat #ab2893  RRID:AB_303388 | IF (1:100) |
| Rabbit polyclonal anti-53BP1 | Cell Signaling Technology | Cat #4937  RRID:AB_10694558 | IF (1:100) |
| Rabbit polyclonal anti-p16 | Santa Cruz Biotechnology | Cat #sc-759  RRID:AB_632105 | WB (1:100) |
| Goat anti-rabbit IgG (H+L) cross-adsorbed polyclonal secondary antibody (Alexa Fluor 488) | Thermo Fisher Scientific | Cat #A-11008, RRID:AB_143165 | IF (1:500) |
| Goat anti-mouse IgG (H+L) highly cross-adsorbed polyclonal secondary antibody (Alexa Fluor 546) | Thermo Fisher Scientific | Cat #A-11030, RRID:AB_2534089 | IF (1:500) |
| Rabbit polyclonal anti-Flag | Cell Signaling Technology | Cat #2368 | WB (1:1000) |
| Mouse monoclonal anti-HA | Cell Signaling Technology | Cat #2367 | WB (1:1000) |
| Mouse monoclonal anti-GAPDH | Santa Cruz Biotechnology | Cat #25778, RRID:AB_10167668 | WB (1:10000) |
| Rabbit monoclonal anti-β-actin | Cell Signaling Technology | Cat #4970, RRID:AB_2223172 | WB (1:1000) |
